# Characteristics of *Lactococcus petauri* GB97 lysate isolated from porcine feces and its *in vitro* and *in vivo* effects on inflammation, intestinal barrier function, and gut microbiota composition in mice

**DOI:** 10.1128/spectrum.01334-23

**Published:** 2023-11-29

**Authors:** Ki-Nam Yoon, Soo-Jeong Lee, Gi Beom Keum, Ki-Young Song, Jong-Heum Park, Beom-Seok Song, Seung Yeob Yu, Jae Hyoung Cho, Eun Sol Kim, Hyunok Doo, Jinok Kwak, Sheena Kim, Jong-Bang Eun, Ju Huck Lee, Hyeun Bum Kim, Ju-Hoon Lee, Jae-Kyung Kim

**Affiliations:** 1 Advanced Radiation Technology Institute, Korea Atomic Energy Research Institute, Jeongeup-si, South Korea; 2 Department of Food Science and Technology, Graduate School of Chonnam National University, Gwangju, South Korea; 3 Department of Food and Animal Biotechnology, Seoul National University, Seoul, South Korea; 4 Department of Agricultural Biotechnology, Seoul National University, Seoul, South Korea; 5 Research Institute of Agriculture and Life Sciences, Seoul National University, Seoul, South Korea; 6 Center for Food and Bioconvergence, Seoul National University, Seoul, South Korea; 7 Department of Animal Resources Science, Dankook University, Cheonan, South Korea; 8 Korean Collection for Type Cultures, Korea Research Institute of Bioscience and Biotechnology, Jeongeup-si, South Korea; Huazhong University of Science and Technology, Wuhan, China

**Keywords:** *Lactococus petauri*, paraprobiotics, lysate, inflammation cytokine, gut microbiota, intestinal barrier function

## Abstract

Weaning in piglet management is important to improve pork production. Controlling gut inflammation and colitis is important in managing growth performance in piglets after weaning. Previously, we found that treatment with a lysate of *Lactococcus petauri* GB97 (LPL97) from porcine feces alleviated lipopolysaccharide-induced inflammatory response in RAW 264.7 cells. Here, we aimed to evaluate the exact role of LPL97 in modulating inflammation, epithelial barrier function, and gut microbiota composition, both *in vitro* and *in vivo*, in an experimental dextran sulfate sodium (DSS)-induced colitis mouse model. Compared to control colitis mice, LPL97 inhibited shortening of the colon, percentage body weight reduction, and mucosal damage. Moreover, LPL97 treatment downregulated the serum levels of pro-inflammatory factors and their mRNA expression in colon tissue but increased the levels of anti-inflammatory cytokine. LPL97 also reduced intestinal permeability by increasing the expression levels of tight junction (TJ) proteins in colon tissues. Additionally, 16S rRNA gene analysis demonstrated the effect of LPL97 treatment on DSS-induced inflammation via microbiome changes in the mouse intestine. At the genus level, the relative abundance of *Mucispirillum*, *Intestinimonas*, *Staphylococcus*, and *Pseudomonas*, which was significantly higher in the DSS-treated group than in the control group, decreased significantly in the LPL97 group compared to that in the DSS group. This study provides evidence that LPL97 can prevent intestinal inflammation and strengthen the integrity of the intestinal barrier by altering the gut microbiota, suggesting its promising potential for prophylaxis and treatment of colitis in pig farming.

**IMPORTANCE:**

Weaning is a crucial step in piglet management to improve pork production. During the weaning phase, disruption of epithelial barrier function and intestinal inflammation can lead to decreased absorption of nutrients and diarrhea. Therefore, maintaining a healthy intestine, epithelial barrier function, and gut microbiota composition in this crucial phase is strategic for optimal weaning in pigs. We isolated a lysate of *Lactococcus petauri* GB97 (LPL97) from healthy porcine feces and evaluated its anti-inflammatory activities, barrier integrity, and gut microbial changes in LPS-induced murine macrophages and DSS-induced colitis mice. We found that LPL97 regulated the immune response by downregulating the TLR4/NF-κB/MAPK signaling pathway both *in vitro* and *in vivo*. Furthermore, LPL97 alleviated the disruption of intestinal epithelial integrity and gut microbiota dysbiosis in colitis mice. This study indicates that LPL97 has the potential to be developed as an alternative feed additive to antibiotics for the swine industry.

## INTRODUCTION

Pork is the most widely eaten meat from domestic animals globally (1). Weaning is an important step in piglet management to improve pork production. This phase involves multiple changes: breast milk replacement by solid feed, separation from the mother sow, and relocation with unfamiliar littermates (2). This stressful environment results in the disruption of epithelial barrier function, upregulation of pro-inflammatory cytokines, and intestinal inflammation, which can lead to decreased nutrient absorption and post-weaning diarrhea (2 – 4). Therefore, maintaining a healthy intestine, epithelial barrier function, and gut microbiota composition in this crucial phase appears strategic for optimal weaning in pigs.

Antibiotics are commonly used in weaning diets to alleviate intestinal inflammation and gut disorders and improve growth performance by preventing the growth of harmful microorganisms, such as enterotoxigenic *Escherichia coli* (ETEC) and *Salmonella* spp. However, the European Union has mandated the use of other substances instead of antibiotics as growth promoters since 2006 owing to the occurrence of resistant pathogenic strains and antibiotic residues in animal products (5). Alternatively, bacterial and yeast probiotics are being used as feed additives to enhance epithelial barrier function and promote immune function and general health (6, 7). However, many safety issues with the use of live microorganisms, particularly in livestock, remain; probiotic bacteria administration to animals with dextran sulfate sodium (DSS)-induced colitis aggravates the condition (8 – 10). Moreover, probiotics may contain antibiotic-resistant genes and be capable of conjugating with native microbes or pathogens to transfer the genes via the plasmid (11). Therefore, it is important to carefully select probiotics for feed additives.

Paraprobiotics are inactivated microbial cells or cell fractions that confer health benefits to the host. Paraprobiotics consist of a wide range of molecules, including surface proteins, lipoteichoic acid, peptidoglycans, and exopolysaccharides (EPSs) (12). Heat-killed cells or fractionated cell components have beneficial health effects (13), and *Lactobacillus rhamnosus* GG and its components (surface layer protein and exopolysaccharide) may effectively ameliorate inflammation in porcine intestinal epithelial cells stimulated by lipopolysaccharide (LPS) (14). Heat-killed probiotics, such as *Enterococcus faecalis*, and fractionated cell components, such as cell wall preparations from *Lactobacillus* spp. and lysate from *L. plantarum* or *L. casei*, enhance host resistance and stimulate innate immune responses (15 – 18).

Gut microbiota has a wide range of functions in the host gastrointestinal tract, including the development of the immune system, protection from pathogens, inflammation, and homeostasis (19 – 21). Both probiotics and paraprobiotics help maintain the health of humans and animals by improving the intestinal microbiome (22 – 24). However, only limited reports exist on paraprobiotics isolated from porcine feces with the ability to prevent LPS-induced inflammation and DSS-induced colonic inflammation. Therefore, the present study was conducted to screen newly isolated paraprobiotic strains from healthy porcine feces to protect against LPS-induced inflammation in RAW 264.7 cell lines. Furthermore, based on the paraprobiotic attributes, *L. petauri* was evaluated for its ability to reduce DSS-induced colitis in mice.

## RESULTS

### GB97 lysate treatment inhibits nitric oxide production in RAW 264.7 cells

**Fig 1:**
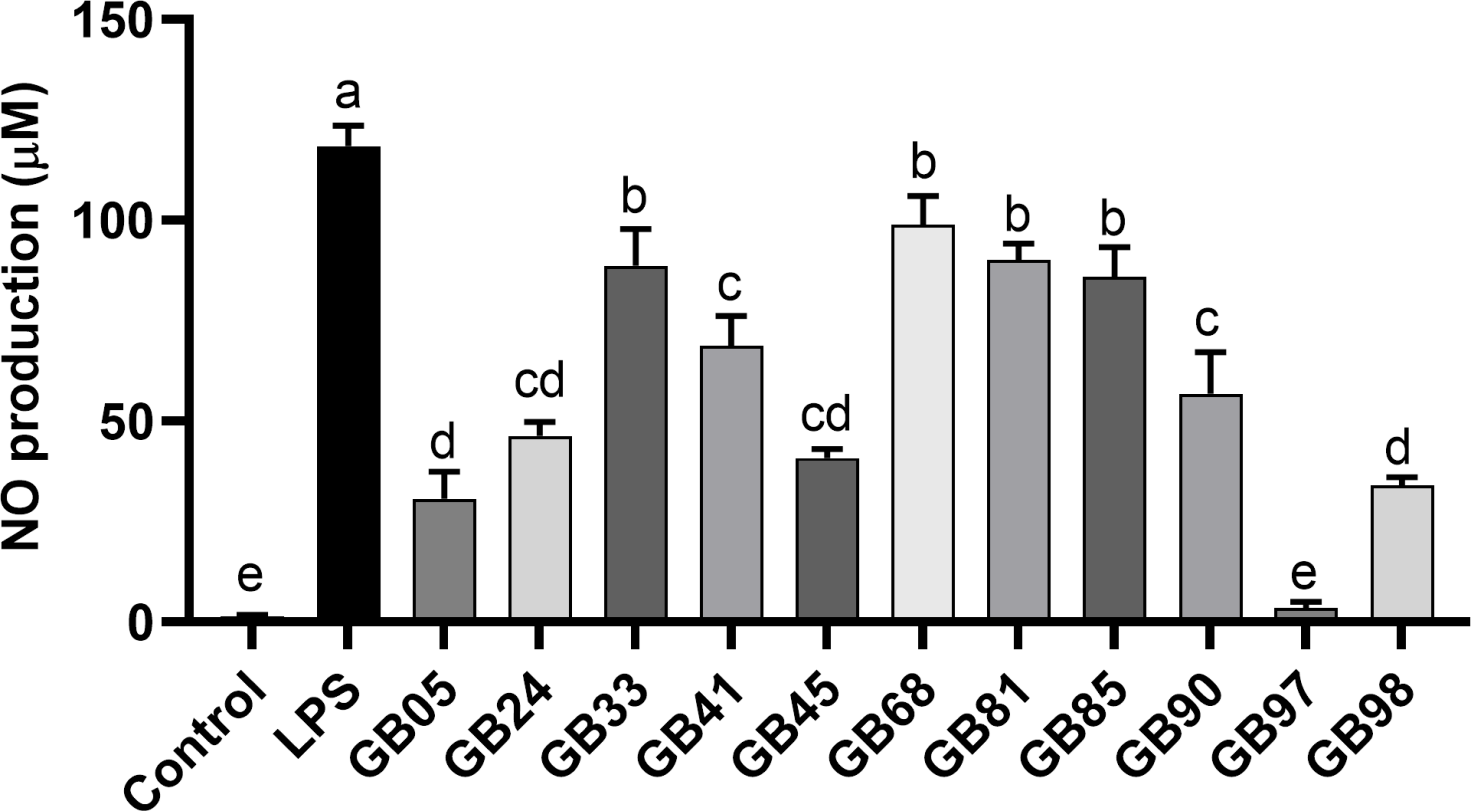
Inhibition of nitric oxide (NO) production on lipopolysaccharide (LPS)-stimulated murine macrophages. Among the 100 gut bacterial lysates, 11 inhibited NO production (GB01, 03, 05, 24, 33, 34, 38, 41, 45, 68, 81, 83, 85, 90, 97, and 98).

Among lysates of the 100 isolated gut bacteria strains, 11 strains (GB05, 24, 33, 41, 45, 68, 81, 85, 90, 97, and 98) showed nitric oxide (NO) production inhibition in LPS-stimulated RAW 264.7 cells (Table 1). Notably, GB33, 68, 81, and 85 lysates demonstrated a reduction of >20% in NO production, and GB05, 24, 45, 90, and 98 lysates showed a reduction of >60% (Fig. 1). The GB97 lysate showed the highest NO production inhibition up to 92%. Therefore, GB97 was selected for further study.

**TABLE 1:** NO production in LPS-induced Raw 264.7 cells treated with GM lysates

No.	NO inhibition	No.	NO inhibition	No.	NO inhibition
GM01	**+^*a*^**	GM35	-	GM69	-
GM02	-^*b*^	GM36	-	GM70	-
GM03	**+**	GM37	-	GM71	-
GM04	-	GM38	**+**	GM72	-
GM05	**+**	GM39	-	GM73	-
GM06	-	GM40	-	GM74	-
GM07	-	GM41	+	GM75	-
GM08	-	GM42	-	GM76	-
GM09	-	GM43	-	GM77	-
GM10	-	GM44	-	GM78	-
GM11	-	GM45	**+**	GM79	-
GM12	-	GM46	-	GM80	-
GM13	-	GM47	-	GM81	**+**
GM14	-	GM48	-	GM82	-
GM15	-	GM49	-	GM83	**+**
GM16	-	GM50	-	GM84	-
GM17	-	GM51	-	GM85	**+**
GM18	-	GM52	-	GM86	-
GM19	-	GM53	-	GM87	-
GM20	-	GM54	-	GM88	-
GM21	-	GM55	-	GM89	-
GM22	-	GM56	-	GM90	**+**
GM23	-	GM57	-	GM91	-
GM24	**+**	GM58	-	GM92	-
GM25	-	GM59	-	GM93	-
GM26	-	GM60	-	GM94	-
GM27	-	GM61	-	GM95	-
GM28	-	GM62	-	GM96	-
GM29	-	GM63	-	GM97	**+**
GM30	-	GM64	-	GM98	**+**
GM31	-	GM65	-	GM99	-
GM32	-	GM66	-	GM100	-
GM33	**+**	GM67	-		
GM34	**+**	GM68	**+**		

^*a*^ Inhibition of nitric oxide production greater than 60%.

^*b*^ No inhibition.

### Genomic characteristics of *L. petauri* GB97

**Fig 2:**
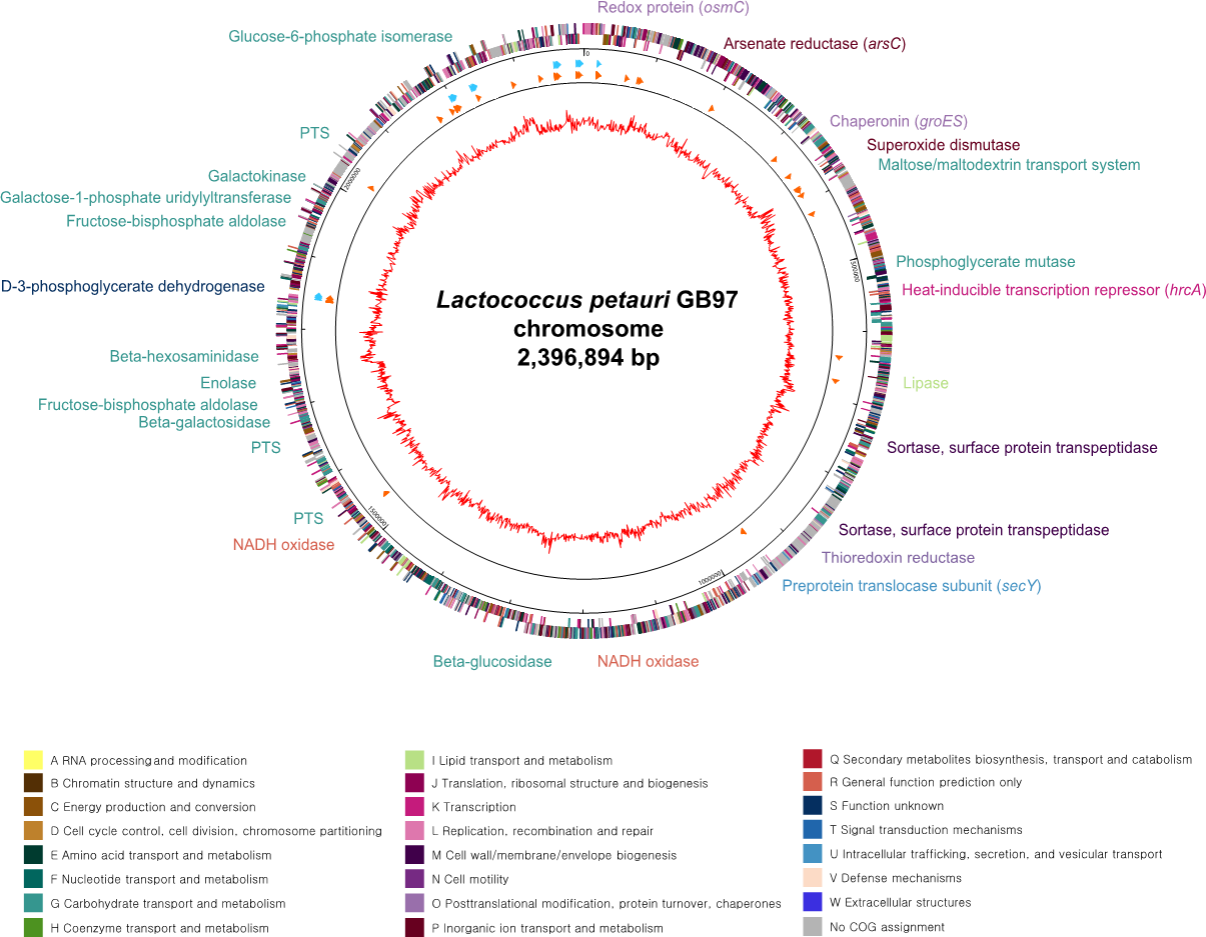
Circular genome map of *L. petauri* GB97. The outer circle indicates the location of all annotated open reading frames (ORFs) in double strands colored by clusters of orthologous groups (COG), and the inner circle with red peaks indicates GC contents. Sky blue arrows, marked between these circles, indicate rRNA operons and orange arrows indicate the tRNAs. The color codes of COG categories are indicated below.

GB97 was identified as *L. petauri* using 16S rRNA sequencing. The complete genome sequence of *L. petauri* GB97 consists of 2,396,894 bp circular double-stranded DNA containing 2,388 open reading frames (ORFs) with a GC content of 37.7% (Fig. 2). It has 65 tRNA genes and 5 rRNA operons with one extra rRNA gene. Metabolic pathway analysis revealed its complete carbohydrate utilization and energy production activities; it has complete glycolysis and citric acid cycles, indicating that it can produce pyruvate and reduced nicotinamide adenine dinucleotide (NADH) via pyruvate catabolism. Additionally, a complete electron transport chain indicated the bioconversion activity of NADH to ATP. The GB97 genome has β-galactosidase and galactose-1-phosphate uridylyltransferase, indicating the efficient digestion and utilization of lactose and galactose. Therefore, this strain may utilize lactose for energy production.

GB97 has oxygen resistance-related genes encoding NADH oxidase (GB97_01569), thioredoxin reductase (GB97_00818), and superoxide dismutase (SOD; GB97_00318) (Fig. 2). NADH oxidase produces hydrogen peroxide (H_2_O_2_) with NADH, which has many toxic properties. To remove this toxic compound, SOD converts oxygen radicals to water, and thioredoxin reductase reduces H_2_O_2_ to water. Therefore, the combination of these three enzymes provides *L. petauri* GB97 with temperate oxygen resistance activity, which is important for its survival in the gut habitat. Arsenic is widely present in various environments, especially water. Given the prevalence of consumption of foods contaminated with low arsenic levels, the resistance activity against its chronic toxicity is important for intestinal bacteria survival in the gut habitat; these arsenic resistance genes and their activities have been frequently detected (25, 26). *L. petauri* GB97 possesses a related gene encoding arsenate reductase (ArsC), which is responsible for reducing arsenate to arsenite. Moreover, in the GB97 genome, more than two copies of sortase enzymes, which are associated with the modification and activation of cell surface proteins for bacterial adhesion to the mucus layer, were detected (27). This adaptation is expected to exert a substantial influence on GB97’s ability to adhere to and interact with the mucus layer in the gut. The GB97 strain has *rmlA* and *rmlB* genes (Table 2) that participate in the O-antigen nucleotide sugar metabolism pathway in gram-negative bacteria (28). However, since *L. petauri* GB97 is gram-positive, *rmlABCD* are predicted to be involved in the polysaccharide synthesis on the bacterial cell wall (29).

**TABLE 2:** Cytokine-specific primer sets^*a*^

No.	Target	Primer sequence (5′−3′)	
		Forward	Reverse
1	β-actin	TGA GCT GCG TTT TAC ACC CT	GCC TTC ACC GTT CCA GTT TT
2	TNF-α	TTC TGT CTA CTG AAC TTC GGG GTG ATC GGT CC	GTA TGA GAT AGC AAA TCG GCT GAC GGT GTG GG
3	IL-6	GTC CTT CCT ACC CCA ATT TCC A	TAA CGC ACT AGG TTT GCC GA
4	IL-1β	GAA AGA CGG CAC ACC CAC CCT	GCT CTG CTT GTG AGG TGC TGA TGT A
5	IL-10	GCT GAG GCG CTG TCA TCG ATT T	GGC CCT GCA GCT CTC AAG TGT
6	TLR4	TTC AGA GCC GTT GGT GTA TC	CCC ATT CCA GGT TAG GTG TTT
7	MyD88	CAC TCG CAG TTT GTT GGA TG	CCA CCT GTA AAG GCT TCT CG
8	iNOS	CCC TTC CGA AGT TTC TGG CAG CAG C	GGC TGT CAG AGC CTC GTG GCT TTG G
9	β-actin	AAT CGT GCG TGA CAT CAA	GCT CGT TGC CAA TAG TGA
10	TNF-α	AGG GTC TGG GCC ATA GAA CT	CCA CCA CGC TCT TCT GTC TAC
11	IL-6	GAG GAT ACC ACT CCC AAC AGA CC	AAG TGC ATC ATC GTT GTT CAT ACA
12	IL-1β	CTG AAC TCA ACT GTG AAA TGC	TGA TGT GCT GCT GCG AGA
13	IL-10	ACA GCC GGG AAG ACA ATA AC	CAG CTG GTC CTT TGT TTG AAA G
14	TLR4	ATC GCC TAT GGT TGT TGA CC	GGT TTC ACG ACT GGA GGT TC
15	MyD88	GCA TGG TGG TGG TTG TTT CTG	GAA TCA GTC GCT TCT GTT GG
16	iNOS	GGC AGC CTG TGA GAC CTT TG	GCA TTG GAA GTG AAG CGT TTC
17	COX-2	GAA TCA TTC ACC AGG CAA ATT G	TCT GTA CTG CGG GTG GAA CA
18	ZO-1	ACT CCC ACT TCC CCA AAA AC	CCA CAG CTG AAG GAC TCA CA
19	Claudin 1	ACT CCC ACT TCC CCA AAA AC	CCA CAG CTG AAG GAC TCA CA
20	Claudin 3	CCT GTG GAT GAA CTG CGT G	GTA GTC CTT GCG GTC GTA G
21	MUC2	GGC CTC ACC ACC AAG CGT CC	TGG GCT GGC AGG TGG GTT CT

^*a*^ 1–8; RAW 264.7 cell primer, 9–21; TNF-α, tumor necrosis factor alpha; IL, interleukin; Mouse primer; -like receptor (TLR) 4; iNOS, inducible nitric oxide synthase; MyD88, myeloid differentiation primary response; COX-2; ZO-1, Zonula occludens-1; MUC2, mucin 2.

### Phylogenetic analysis

**Fig 3:**
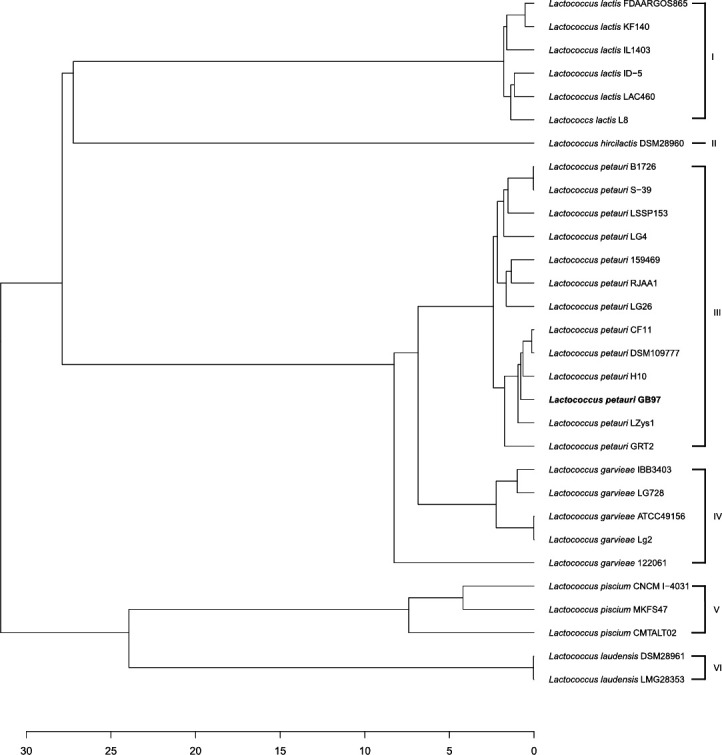
Phylogenetic analysis of *L. petauri* GB97 and related species. A phylogenetic tree was generated based on average nucleotide identity values. Roman numerals indicate the six different species of *Lactococcus. L. petauri* GB97 is indicated in bold.

A phylogenetic average nucleotide identity (ANI) tree was constructed with 30 *Lactococcus* strains (Fig. 3), revealing six different groups associated with *Lactococcus* species. Group III showed two different subgroups. *L. petauri* GB97 belongs to the second subgroup of group III, and it is closely related to CF11 (99.38% similarity), DSM109777 (99.70%), and H10 (98.97%). A previous genome analysis study of *L. petauri* CF11 showed that it is adapted to the healthy human gut environment, similar to the strain GB97 (30). However, the other two strains have not been genomically and experimentally characterized. Therefore, this study may help understand the representative characteristics of *L. petauri*.

### Safety assessment of *L. petauri* GB97

#### Bioinformatic-based safety assessment

To ensure the safety of *L. petauri* GB97 use, virulence factor database (VFDB) analysis was performed to detect any virulence factors in the genome. However, no virulence factor was detected. Additionally, in a comparative analysis using Pathogen Finder between pathogenic and non-pathogenic bacteria using whole genome sequence data (31), *L. petauri* GB97 was predicted to be a non-pathogen, suggesting that *L. petauri* GB97 is a non-toxigenic strain. Comprehensive antibiotic resistance database (CARD) analysis for detecting antibiotic resistance genes in the genome revealed that the *L. petauri* GB97 genome contains no antibiotic resistance genes. Additional horizontal gene transfer (HGT) analysis using the ResFinder software showed that this genome has little or no possibility of acquired resistance by HGT events, supporting the lack of pathogenic characteristics in GB97.

#### Hemolytic activity

**Fig 4:**
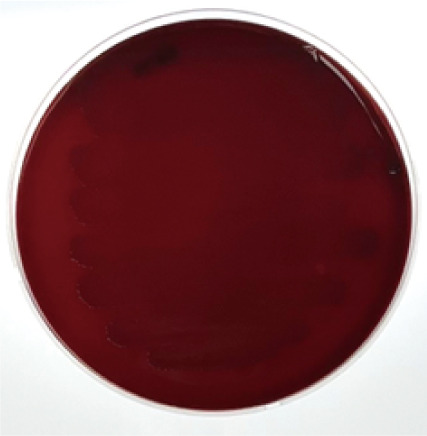
Hemolytic activity of *L. petauri* GB97.

The hemolytic activity of the GB97 strain was evaluated on a blood agar plate, and *L. petauri* GB97 showed no hemolytic activity (Fig. 4).

#### Cytotoxicity

**Fig 5:**
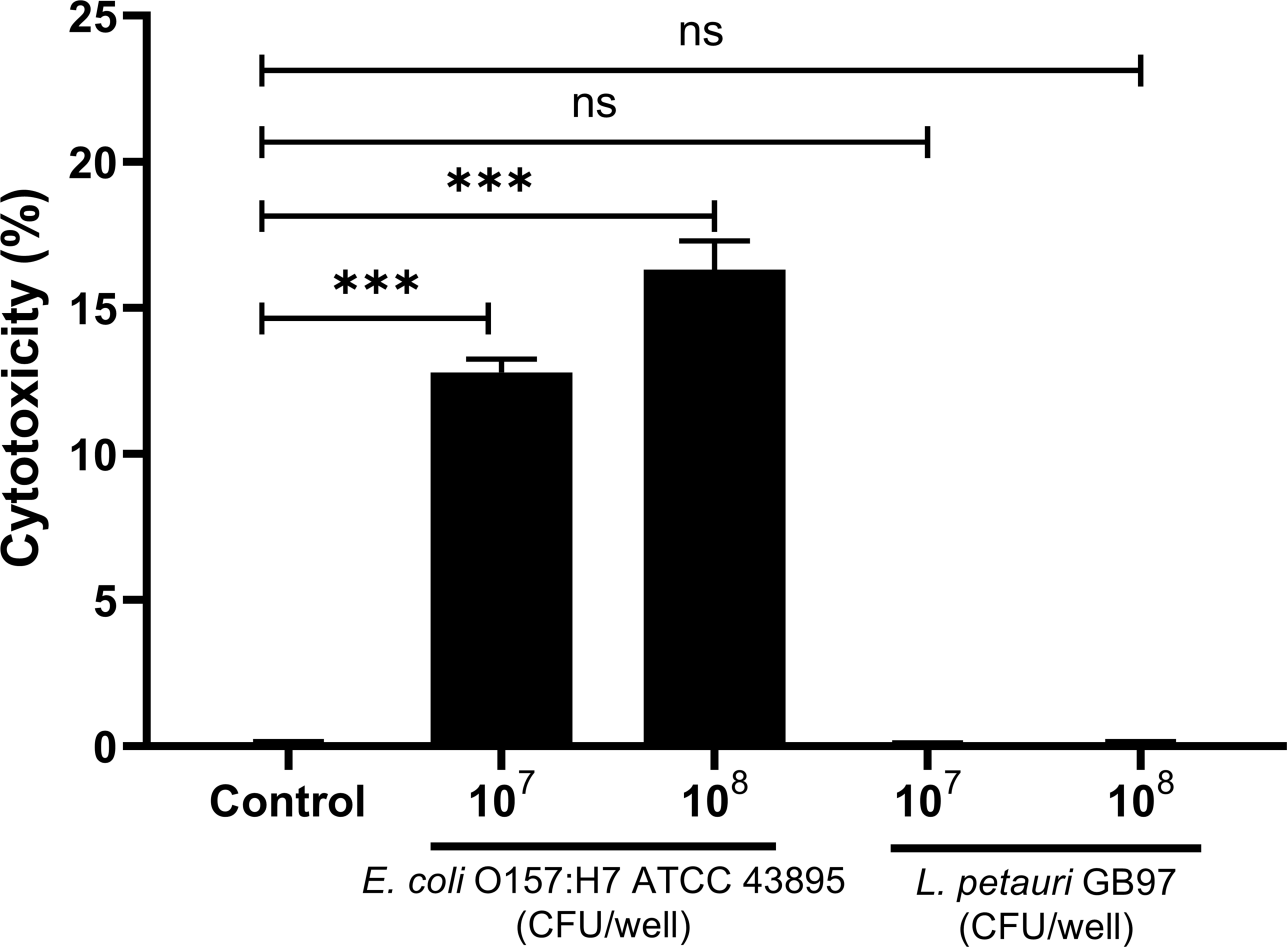
Cytotoxic effects of *L. petauri* GB97 on Caco-2 cells after 24 h of treatment. All data are expressed as the mean ± SD. ***P < 0.001, compared to the control group by Student’s *t* test; ns, not significant.

After treatment with *L. petauri* GB97, there was no statistically significant difference in cell cytotoxicity between the experimental and control groups (*P* > 0.05); however, cell cytotoxicity was significantly increased after treatment with *E. coli* O157:H7 ATCC 43895 (Fig. 5), indicating that *L. petauri* GB97 did not cause notable cell damage.

### *Ex vivo* anti-inflammation

**Fig 6:**
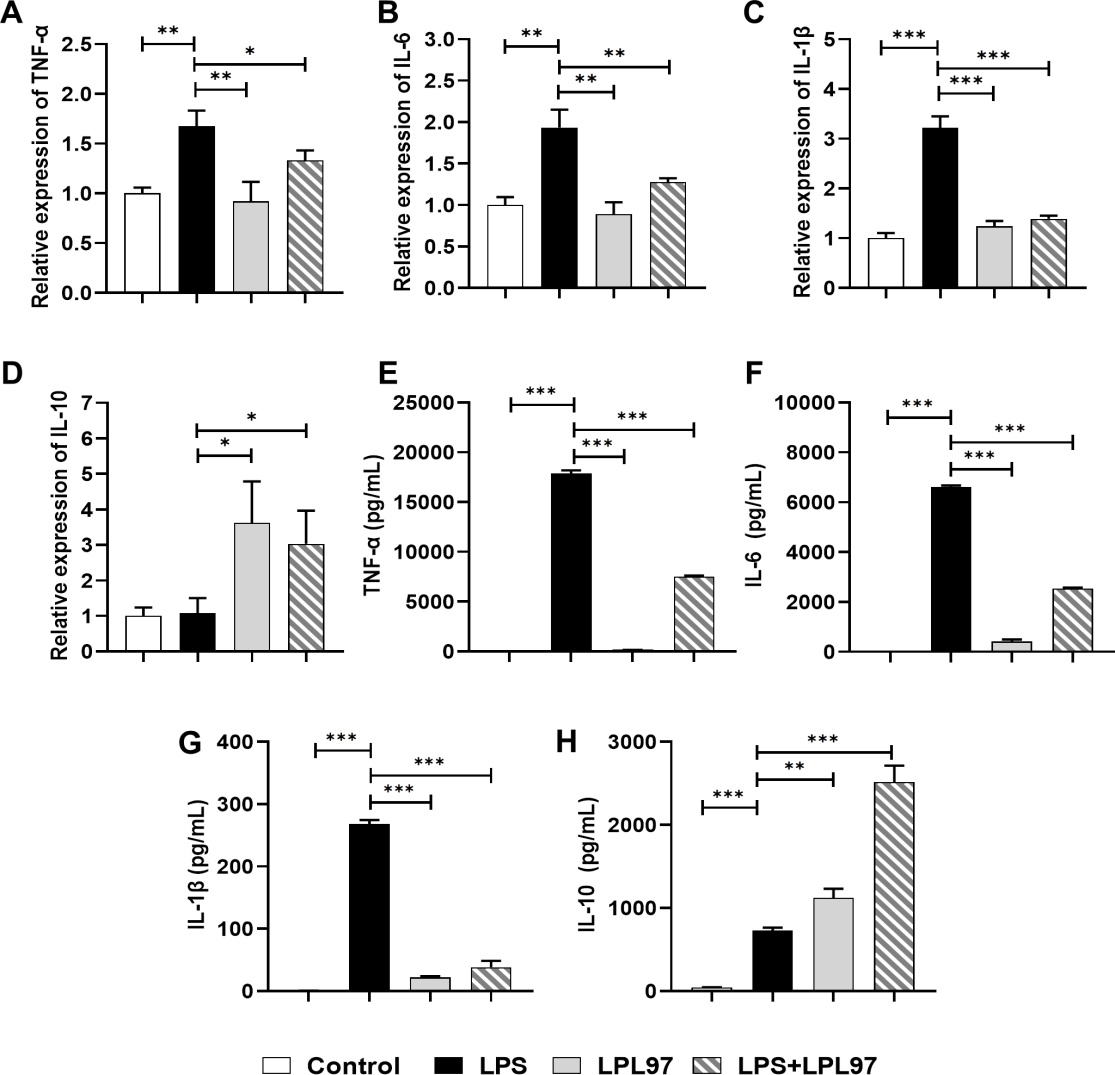
Relative mRNA expression level of inflammation cytokines in RAW 264.7 cells. (**A**) TNF-α, (**B**) IL-6, (**C**) IL-1β, and (**D**) IL-10. (**E–H**) Inflammation-related cytokine of cell-free supernatant in raw 264.7 cells. Control: treated with phosphate-buffered saline; LPS: treated with lipopolysaccharide (100 ng/well); LPL97: pre-treated with *L. petauri* GB97 cell lysate (50 µg/well); LPL97+LPS: pre-treated with LPL97 (50 µg/well), and then post-treated with LPS (100 ng/well). All data are expressed as the mean ± SD. *P < 0.05, **P < 0.01, ***P < 0.001 compared to the LPS group using Student’s *t* test.

The cytotoxicity of LPL97 was evaluated using a cell viability assay (Fig. S2). LPL97 treatment did not significantly affect the viability of RAW 264.7 cells within a specific concentration range. Changes in the mRNA expression levels of pro-inflammatory cytokines, including tumor necrosis factor-alpha (TNF-α), interleukin-1 beta (IL-1β), and L-6 and an anti-inflammatory cytokine (IL-10) were assessed in LPS-stimulated RAW 264.7 cells using real-time quantitative polymerase chain reaction (RT-qPCR). Compared to that in control, the mRNA expression of pro-inflammatory cytokines was significantly decreased in both LPL97− and LPL97+LPS-treated groups (Fig. 6A through C). Conversely, LPL97 treatment upregulated the transcription level of the anti-inflammatory cytokine (Fig. 6D). LPL97− and LPL97+LPS-treated groups showed similar patterns in enzyme-linked immunosorbent assay (ELISA) (Fig. 6E through H). These findings suggest that LPL97 treatment contributes to a decrease in the mRNA expression of pro-inflammatory cytokines and an increase in anti-inflammatory cytokine production, indicating its potential anti-inflammatory properties. The consistency observed between the mRNA expression analysis and ELISA further strengthens the reliability of the results.

### *Ex vivo* TLR4//NF-κB and mitogen-activated protein kinase signaling pathway

**Fig 7:**
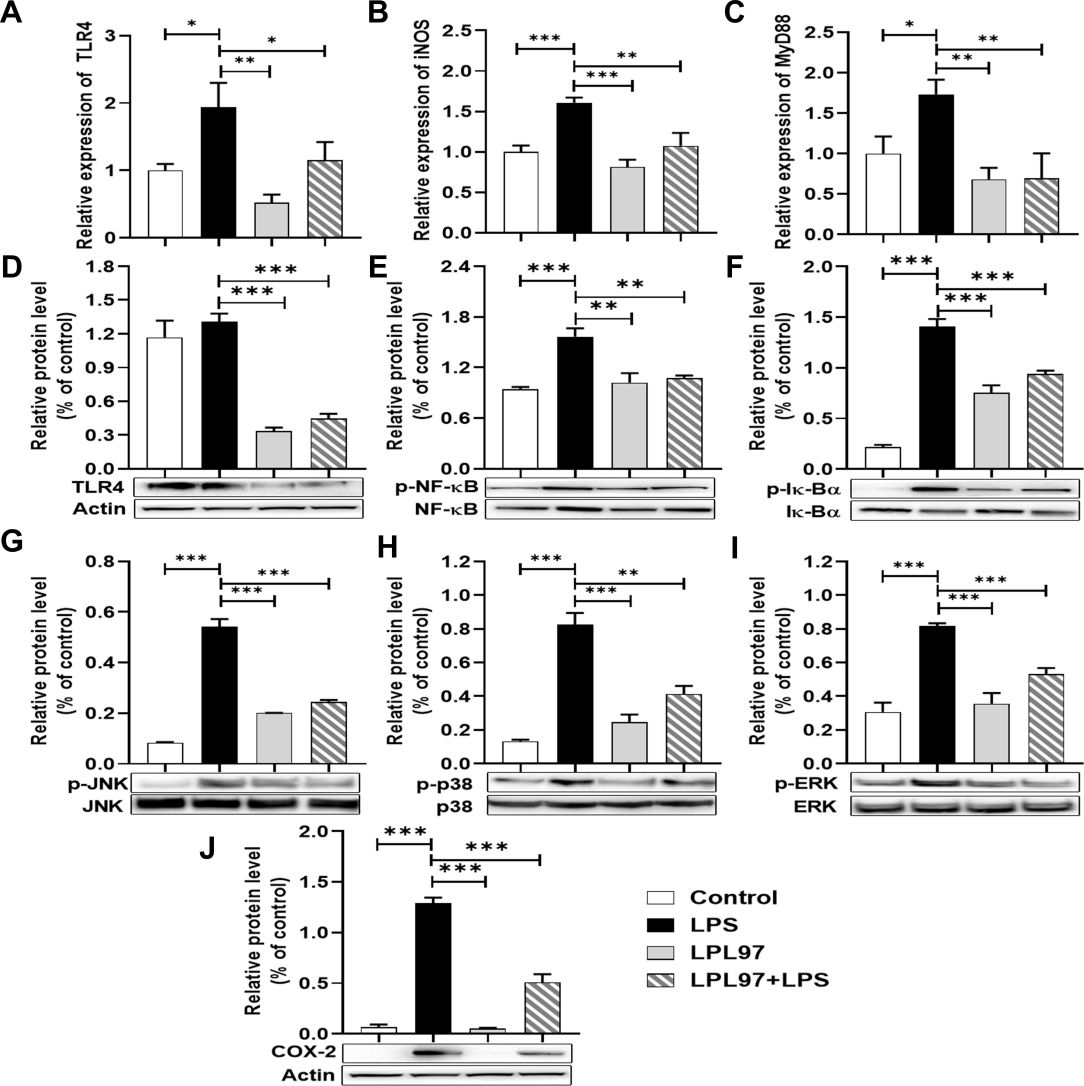
Relative mRNA expression and protein levels of inflammatory cytokines in RAW 264.7 cells. (**A**) Toll-like receptor (TLR) 4, (**B**) inducible nitric oxide synthase (iNOS), (**C**) myeloid differentiation primary response 88 (MyD88), (**D**) TLR4, (**E**) nuclear factor kappa-light-chain-enhancer of activated B cells (NF-κB), (**F**) Iκ-Bα, (**G**) JNK, (**H**) p38, (**I**) ERK, and (**J**) cyclooxygenase-2 (COX-2). Control: treated with phosphate-buffered saline; LPS: treated with lipopolysaccharide (100 ng/well); LPL: pre-treated with *L. petauri* GB97 cell lysate (LPL, 50 µg/well); LPL+LPS: pre-treated with LPL (50 µg/well), and then post-treated with LPS (100 ng/well). Data are expressed as the mean ± SD. *P < 0.05, **P < 0.01, ***P < 0.00 compared to the LPS group using Student’s *t* test.

To elucidate inflammatory response modulation by LPL, changes in mRNA and protein expression of Toll-like receptor 4 (TLR4), myeloid differentiation primary response 88 (MyD88), nuclear factor kappa-light-chain-enhancer of activated B cells (NF-κB), and mitogen-activated protein kinase (MAPK) were analyzed. LPS treatment significantly increased TLR4, MyD88, and inducible NO synthase (iNOS) mRNA expression levels compared to the control, and these increases were significantly reduced by LPL97 and LPL97+LPS treatments (Fig. 7A through C).

Western blot analysis demonstrated similar patterns of expression of TLR4, NF-κB, Iκ-bα, and MAPKs (p38, ERK, and JNK) in all experimental groups (Fig. 7D through J), suggesting that LPL97 in murine macrophages leads to decreased pro-inflammatory cytokine levels by modulating the TLR4/NF-κB and MAPK pathways. The downregulation of TLR4, MyD88, and iNOS at the mRNA level, as well as the modulation of key proteins involved in the TLR4/NF-κB and MAPK pathways, further support the anti-inflammatory effects of LPL97.

### LPL97 treatment alleviates colitis *in vivo*

**Fig 8:**
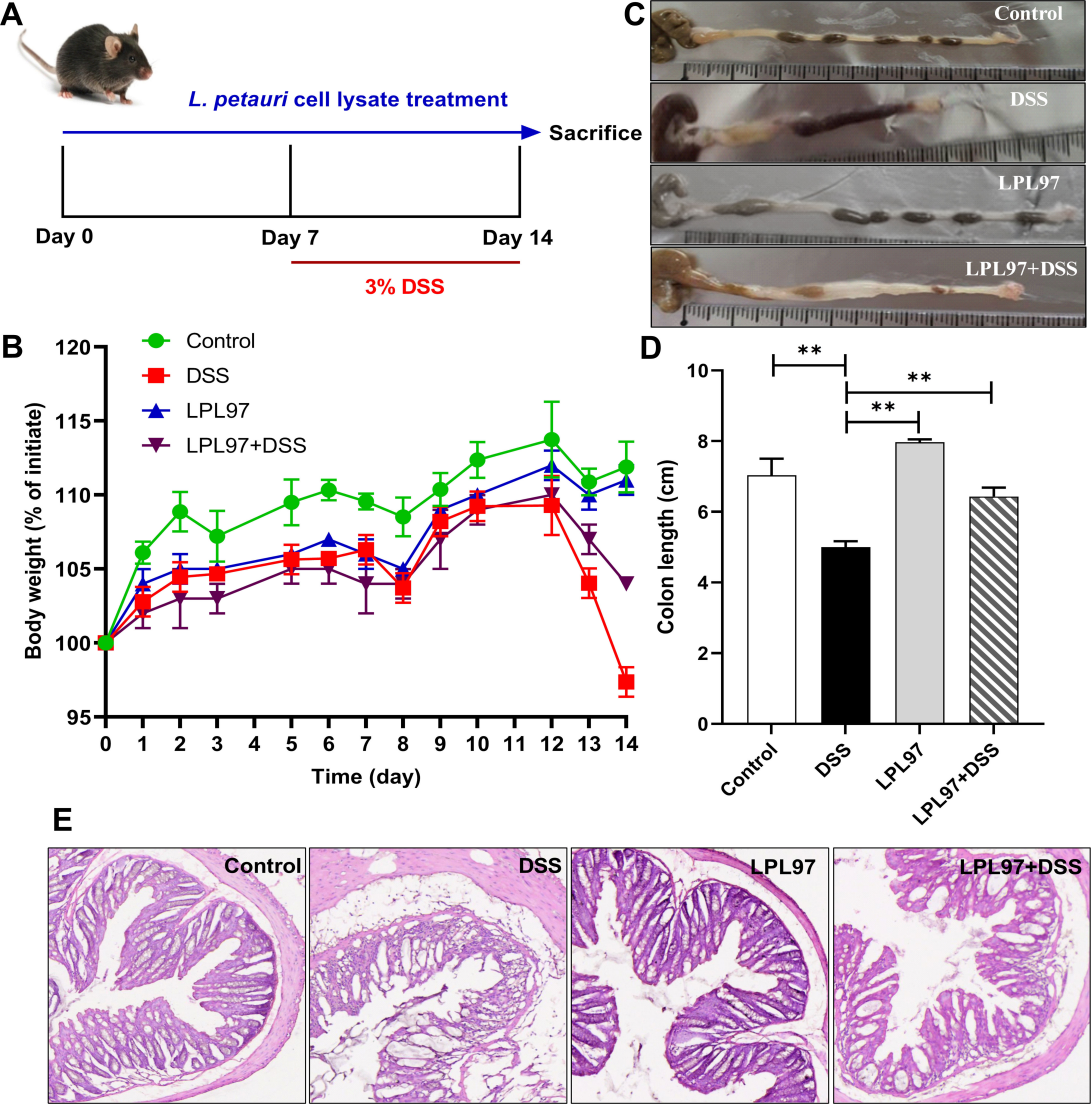
Effects of LPL97 on DSS-induced colitis mice: (**A**) Schematic diagram of the animal experiment. Control group orally gavaged once daily with 100 µL of PBS (0–2 weeks); DSS group received PBS in week 1, but during week 2, mice were given 3% DSS in drinking water; LPL97 groups gavaged once daily with 100 µL of *L. petauri* GB97 lysate (10^9^ CFU/mL in PBS) for 2 weeks; LPL97+DSS groups administered once daily with 100 µL of LPL in week 1, but during week 2, mice were given LPL97 and 3% DSS. (**B**) Body weight of mice. (**C**) A photograph of the measurement and comparison of colon lengths. (**D**) Average colon lengths (cm) in the groups. (**E**) Hematoxylin and eosin staining of representative histological sections of colons from the groups (200× magnification). All data are shown as the mean ± standard deviation (SD). Statistical significance was calculated using Student’s two-tailed *t* test (*P < 0.05, **P < 0.01).

To investigate the effect of LPL97 on colonic inflammation, various parameters, including body weight, colon length, and histological analysis, were assessed in colitis mice. DSS treatment resulted in a significant decrease in body weight compared to the control group (Fig. 8B). However, the LPL97+DSS group showed protective effects against weight loss. Colon length was significantly reduced in the DSS group compared to that in the control group (*P* < 0.01) but was significantly (*P* < 0.01) longer in the LPL97 treatment groups (LPL97, LPL97+DSS) (Fig. 8C and D). Histological analysis of the colon section is shown in Fig. 8E. In the control group, the intestinal epithelium was intact, and the crypt structure was normal with no ulcers. The DSS group showed destruction of the epithelial structure and severe mucosal damage. However, LPL97 administration effectively alleviated colon tissue lesions. These findings suggest that LPL97 treatment alleviates DSS-induced colitis, as evidenced by the improvement in body weight, colon length, and histological observations.

### LPL97 treatment mediates anti-inflammatory response in colitis *in vivo*

**Fig 9:**
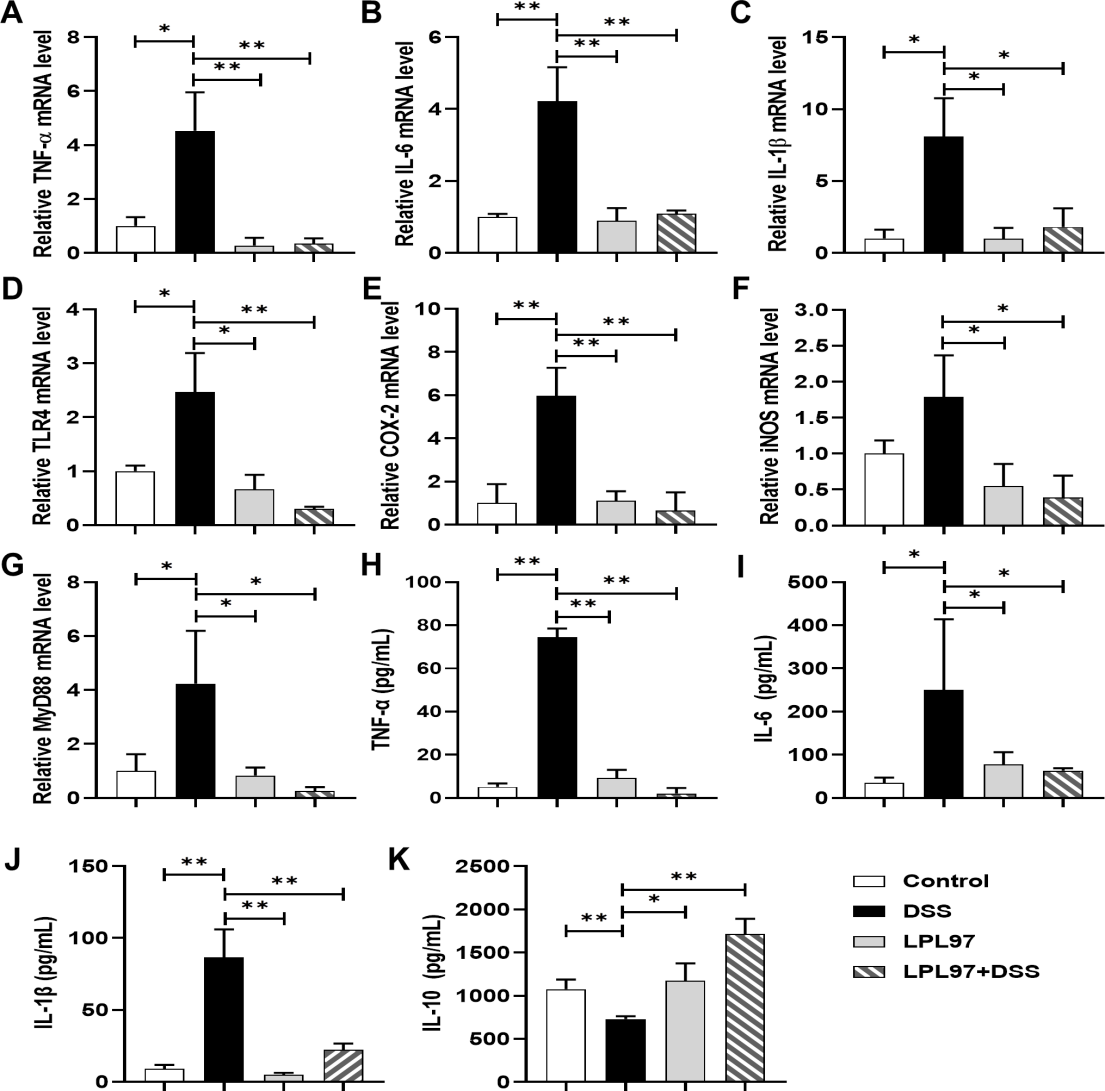
Effects of LPL97 on anti-inflammatory activity in colitis mice. (**A–C**) mRNA expression of the pro-inflammatory cytokines, tumor necrosis factor-alpha (TNF-α), interleukin (IL)-1β, and IL-6. (**D–G**) mRNA expression of toll-like receptor (TLR) 4, inducible nitric oxide synthase (iNOS), toll-like receptor (TLR) 4, and myeloid differentiation primary response 88 (MyD88),. (**H–K**) Secretion of the inflammatory cytokines in serum was determined using ELISA. The data present the mean ± standard deviation (SD). Statistical significance was calculated using Student’s two-tailed *t* test (*P < 0.05, **P < 0.01). Control: oral gavage with PBS; DSS: 3% dextran sulfate sodium; LPL97: *L. petauri* GB97 cell lysate (10^9^ CFU/day); LPL97+DSS: 3% DSS plus LPL97 (10^9^ CFU/day).

To investigate the inflammatory regulation in DSS-induced colitis, the mRNA expression of pro-inflammatory cytokines (TNF-α, IL-1β, and IL-6), iNOS, and cyclooxygenase-2 (COX-2) in the colon tissue of DSS-induced C57BL/6 mice was determined using RT-qPCR. The DSS group showed a significant increase in the mRNA expression of the pro-inflammatory cytokines, iNOS, and COX-2 compared to the control group, which was downregulated by LPL97 treatment. LPL97 also upregulated the expression of the anti-inflammatory cytokine (Fig. 9A through C). To elucidate the inflammatory regulation, mRNA and protein expression related to the TLR4, MyD88, and NF-κB signaling pathways were assessed. DSS treatment significantly increased TLR4, COX-2, iNOS, and MyD88 mRNA expression levels compared to the control, whereas LPL97 and LPL97+DSS treatments significantly reduced them (9D through G). Moreover, LPL97 treatment reduced TNF-α, IL-1β, and IL-6 levels and IL-10 secretion in the serum (Fig. 9H through K).

**Fig 10:**
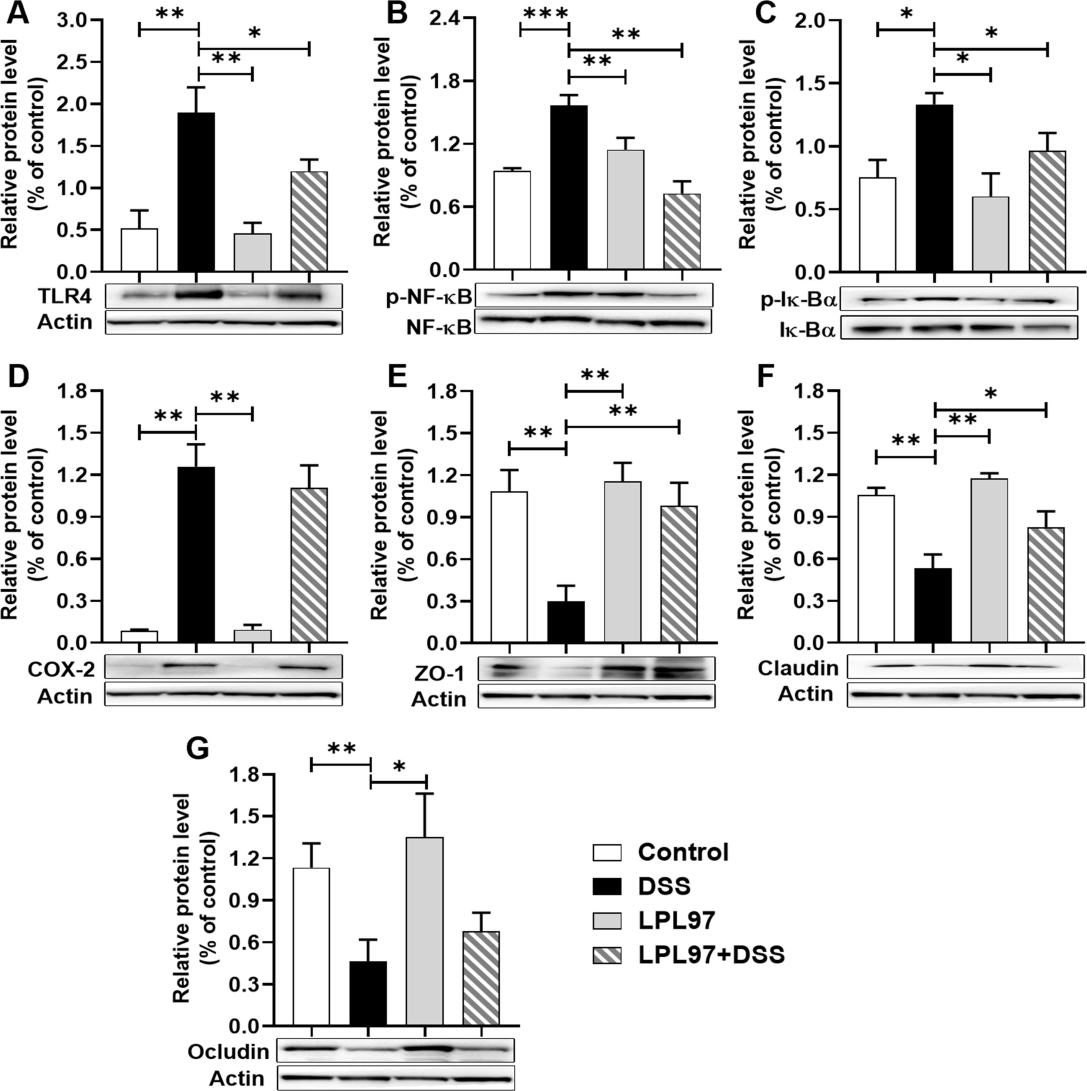
Relative level of inflammation-related and tight junction proteins in colon tissues of colitis mice. (A) Toll-like receptor (TLR) 4, (B) Nuclear factor kappa B (NF-κB), (C) Iκ-Bα, (D) cyclooxygenase (COX)-2, (E) Zonula occludens-1 (ZO-1), (F) Claudin, and (G) Occludin. Control: treated with phosphate-buffered saline; LPS: treated with lipopolysaccharide (100 ng/well); LPL97: pre-treated with *L. petauri* GB97 cell lysate (LPL97, 50 µg/well); LPL97+LPS: pre-treated with LPL97 (50 µg/well), and then post-treated with LPS (100 ng/well). The actin bands depicted in each panel were derived from the same gel, utilizing the same loading control (actin) to consistently compare across varied conditions. Panels (A and D), as well as (E, F, and G), present results extracted from the same experiment. Data are expressed as the mean ± SD. Significance compared to the LPS group by Student’s *t* test (*P < 0.05, **P < 0.01, ***P < 0.001).

Western blot analysis showed similar expression patterns of TLR4, NF-κB, and Iκ-bα in all groups (Fig. 10A through D), further supporting the beneficial effects of LPL97 in the DSS-induced colitis mouse model. The consistent expression patterns of TLR4, NF-κB, and Iκ-bα suggest that LPL97 treatment may modulate the activation of the TLR4/NF-κB signaling pathway, which is associated with regulating inflammatory responses. The protective effects observed in the DSS-induced colitis model further emphasize the potential of LPL97 as a therapeutic agent for inflammatory bowel diseases. LPL97 treatment promotes the secretion of the anti-inflammatory cytokine IL-10, further contributing to regulating the inflammatory response.

### Recovery of colon tight junction in the colitis mouse model

**Fig 11:**
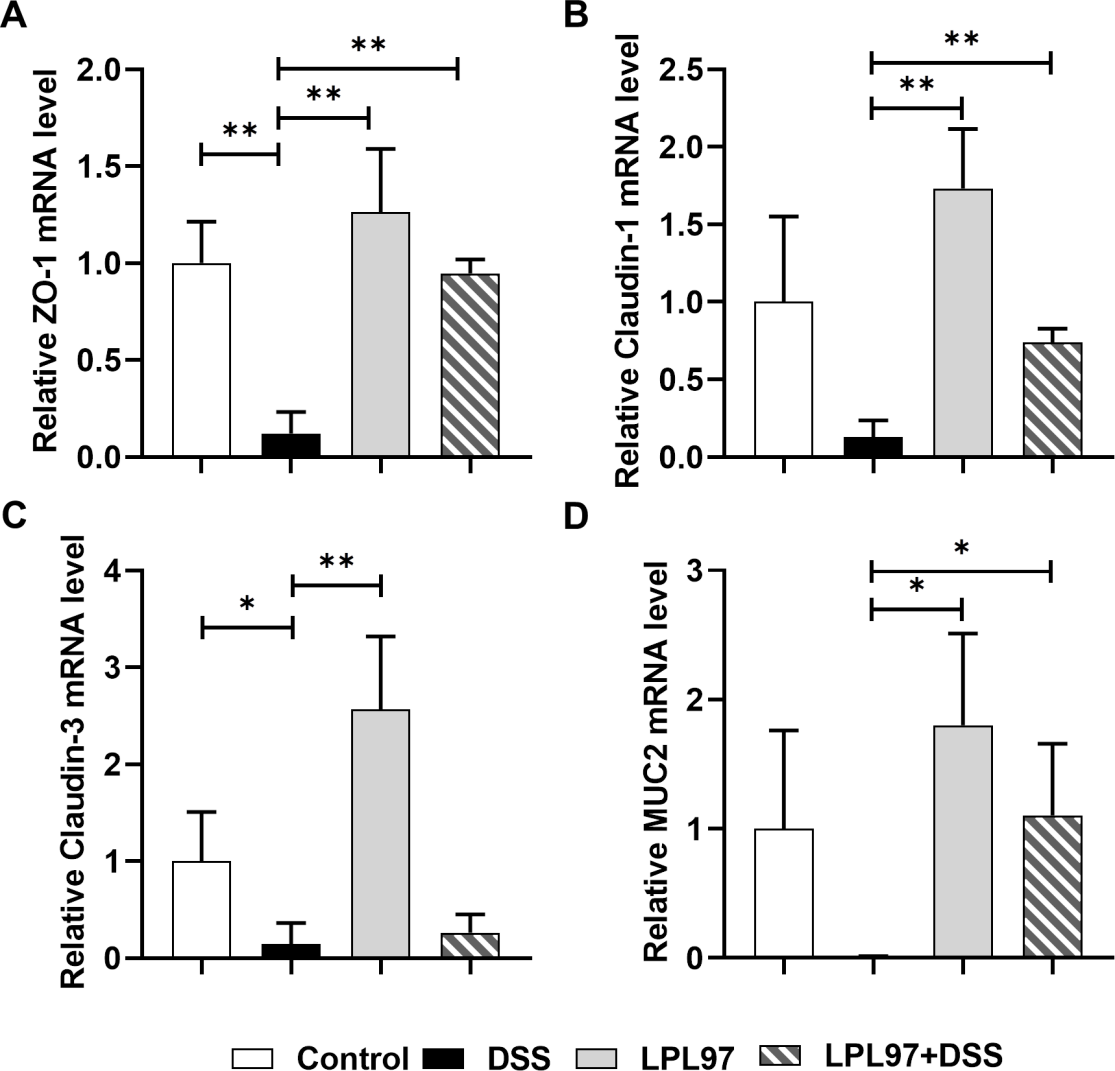
Relative mRNA expression of tight junction and mucin in colon tissues. (**A**) mRNA expression of Zonula occludens-1 (ZO-1). (**B**) mRNA expression of Claudin-1. (**C**) mRNA expression of Claudin-3. (**D**) mRNA expression of mucin 2 (MUC2). The data present the mean ± standard deviation (SD). Statistical significance was calculated using Student’s two-tailed *t* test (*P < 0.05, **P < 0.01). Control: oral gavage with phosphate-buffered saline; DSS: 3% dextran sulfate sodium; LPL97: *L. petauri* cell lysate (10^9^ CFU/day); LPL97+DSS: 3% DSS plus LPL97 (10^9^ CFU/day).

Weakened tight junctions can lead to increased intestinal permeability and decreased barrier function, which can lead to gut disorders, such as irritable bowel syndrome (IBD) in DSS-induced colitis (32). We investigated the effects of LPL97 on the expression of tight junction proteins (ZO-1, Claudin-1, and Occludin) and mucin (MUC2) using both western blotting and RT-qPCR. Compared to the control, DSS treatment significantly decreased the mRNA expression of ZO-1, Claudin-1, Claudin-3, and MUC2 (Fig. 11). Oral administration of LPL97 recovered the mRNA expression of the tight junction proteins, which tended to be higher than that in the control group. Similarly, western blot analysis showed the same patterns of expression in all groups, demonstrating that LPL97 treatment effectively upregulated mucus and tight junction proteins in DSS-induced mice (Fig. 10E through G), thereby enhancing intestinal barrier function and reversing intestinal tissue damage.

### Gut microbiota modulation

**Fig 12:**
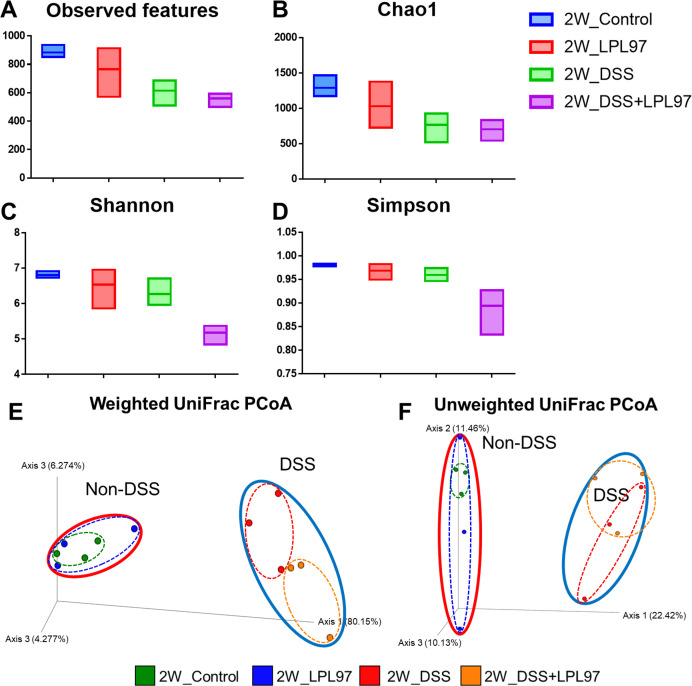
Box plots of the alpha diversity indices in mouse gut microbiomes treated with lysate of *Lactococcus petauri* GB97 (LPL97) and dextran sulfate sodium (DSS). Species richness was measured using (**A**) observed features and (**B**) Chao1 diversity indices. Species evenness was measured using (**C**) Shannon and (**D**) Simpson diversity indices. Each box represents the interquartile range (IQR) between the 25th and 75th percentile, as the horizontal line inside the box indicates the median value. Whiskers denote the lowest and the highest values within 1.5 times from the 25th and 75th quartiles, respectively. Boxes are colored according to the treatment group, as shown in the legend. 2W indicates week 2. Principal coordinate analysis (PCoA) plots of LPL97 and DSS treatment in weeks. At week 2, the non-DSS treated (left; red oval) and DSS treated groups (right; blue oval) were significantly clustered based on unweighted (**E**) and weighted (**F**) UniFrac distance metrics (unweighted: *P* = 0.001, *R*: 0.633333; weighted: *P* = 0.001, *R*: 0.708333).

**Fig 13:**
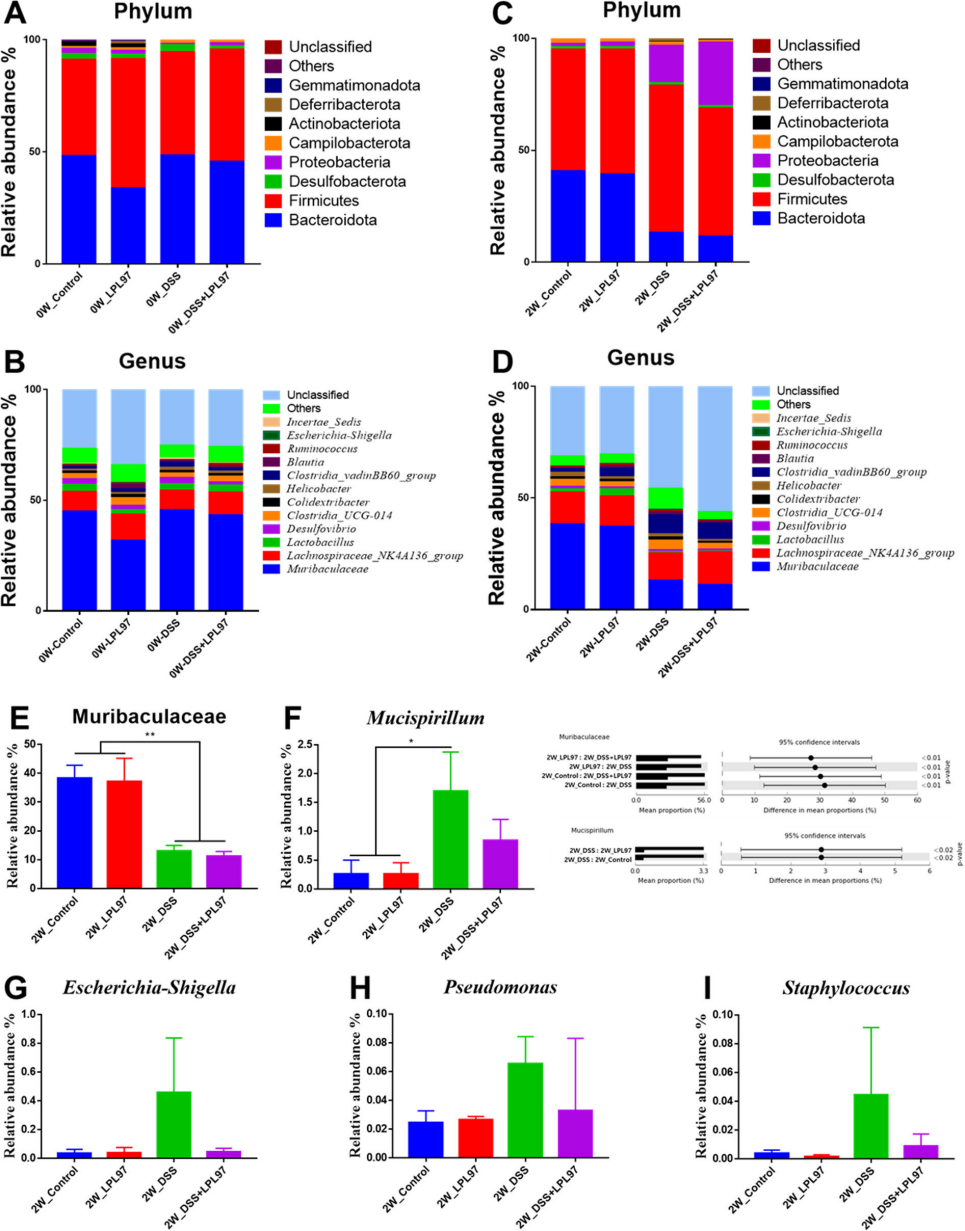
Mouse gut microbiota composition at the phylum and genus level with lysate after treatment with lysate of *Lactococcus petauri* GB97 (LPL97) and dextran sulfate sodium (DSS) Bar plots show the relative abundance of each mouse group taxa at (**A and B**) week 0 and (**C and D**) week 2 at the phylum and genus level. The bar plot (**E–I**) identifying the different taxon between control, LPL97, and DSS treatment. All four groups at the genus (**E–I**) level at week 2 were examined. All genus showed significantly different (** *P* < 0.01; ***P < 0.001; ****P < 0.0001).

Total DNA was extracted from mouse fecal samples, and specific primers for V5 to V6 hypervariable regions of the 16S rRNA genes were used for DNA amplification and sequenced through PCR. A total of 6,610,197 reads were produced after Illumina MiSeq sequencing, which ranged from 83,147 to 421,895 reads per sample. Upon confirming the quality score (Phred scores) of all samples, the base above Q30 per sample accounted for an average of 73.2% from 70.2% to 74.2%. Further data filtering was performed to remove low-quality or uninformative data and improve the downstream statistical analysis, which resulted in 2,642,968 frequency from all the samples, with an average frequency of 110,124 per sample. The diversity of microbial communities in the mouse gut was measured through alpha diversity analysis and derived as species richness (observed features and Chao1) and species evenness (Shannon and Simpson) indices. At week 2, the species richness and evenness index mean values were significantly lower in the DSS (observed features: 615.3 ± 92.79, Chao1: 767.5 ± 217.76, Shannon: 6.27 ± 0.39, and Simpson: 0.96 ± 0.014) and DSS + LPL97 (observed features: 559.3 ± 51.63, Chao1: 703.9 ± 146.88, Shannon: 5.2 ± 0.29, and Simpson: 0.894 ± 0.053) groups than in the control group (observed features: 883 ± 47.03, Chao1: 1289.4 ± 155.04, Shannon: 6.8 ± 0.1, and Simpson: 0.98 ± 0.003) (*P* < 0.05, Fig. 10A through D). However, the LPL97 group (observed features: 766 ± 175.58, Chao1: 1030.4 ± 327.86, Shannon: 6.54 ± 0.59, and Simpson: 0.969 ± 0.017) showed no significant differences, indicating that LPL97 did not significantly influence the microbiota (Fig. 12A through D). Additionally, the principal coordinate analysis (PCoA) plot showed that the microbial composition of the DSS treatment (DSS, DSS + LPL97) and non-treatment groups (control, LPL97) was significantly different in week 2, which was further confirmed by analysis of similarities (ANOSIM) from the weighted and unweighted UniFrac distance results (*P* < 0.05, Fig. 12E and F). Specifically, within the DSS treatment group, the DSS and DSS + LPL97 groups exhibited distinct clustering patterns in the weighted UniFrac analysis. Conversely, within the non-treatment group, no difference was observed according to LPL97 feeding in both weighted and unweighted UniFrac analysis. Taxonomic classification of mouse microbiota according to treatment was performed, which showed the relative abundance at the phylum and genus levels among the four groups in weeks 0 and 2. The most common phyla in all groups at weeks 0 and 2 were Bacteroidota and Firmicutes (Fig. 13A and C). At week 0, the relative abundance of Bacteroidota and Firmicutes in each group was 48.1%, 43.2% (control), 33.7%, 57.6% (LPL97), 48.5%, 45.9% (DSS), and 45.7%, 49.9% (DSS + LPL97), respectively. The relative abundance of Proteobacteria was 2.2%, 1.7%, 0.7%, and 1.3% in the control, LPL97, DSS, and DSS + LPL97 groups, respectively. At week 2, Firmicutes abundance increased compared to that at week 0 in all groups except for the LPL97 groups, while Bacteroidota abundance decreased (Bacteroidota, Firmicutes; control: 40.8%, 54.2%; LPL97: 39.1%, 55.9%; DSS: 13.3%, 65.5%; DSS + LPL97: 11.6%, 57.1%). In the DSS and DSS + LPL97 groups, Proteobacteria was predominant compared to that in the control and LPL97 groups (control: 1.4%; LPL97: 2.1%; DSS: 16.7%; DSS + LPL97: 28.2%). These results are similar to those of previous studies on mouse gut microbiota, indicating that Bacteroidota and Firmicutes are generally the major phyla in both control and DSS-treated mice.

**Fig 14:**
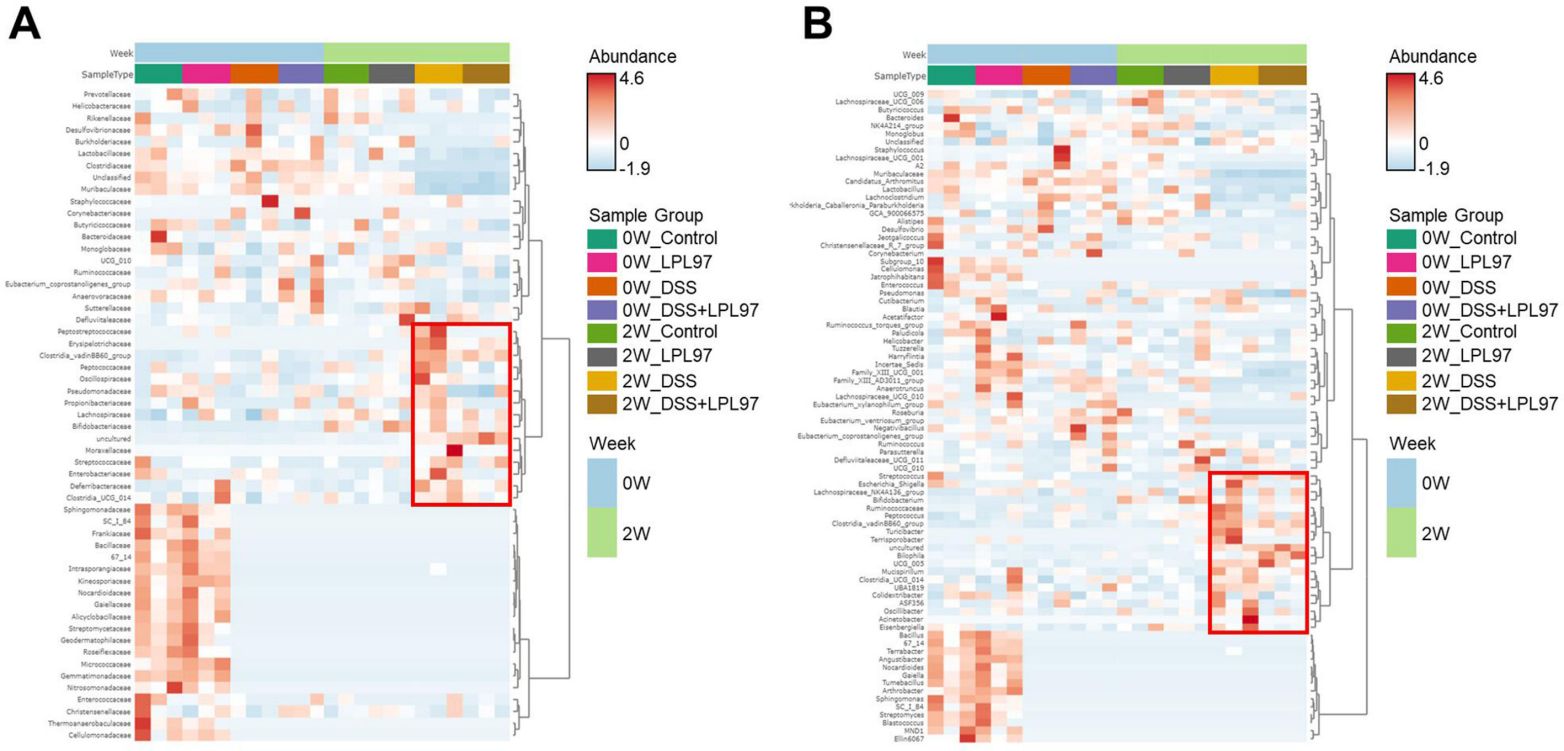
Hierarchical clustering heatmap at the family and genus levels using Ward and Euclidean parameters. In the figure, red represents high abundance and blue represents low abundance. The vertical axis represents the taxonomy level, and the horizontal axis represents the aggregated individuals according to the treatment. The red rectangle represents the unique cluster that showed the higher relative abundances of taxa in the dextran sulfate sodium (DSS) group compared to those in the DSS + LPL97 (lysate of *Lactococcus petauri* GB97) group.

During DSS and LPL97 treatments for 2 weeks, variations between each group (control, LPL97, DSS, and DSS + LPL97) were driven by alterations in the relative abundance of Muribaculaceae bacterium, Lachnospiraceae NK4A136 group, *Lactobacillus*, *Desulfovibrio*, Clostridia UCG 014, *Blautia*, and Clostridia vadinBB60. (Fig. 13B and D). Muribaculaceae bacterium showed a relative abundance of 41.3% at week 0 in the fecal microbiome of all mouse groups. Upon DSS treatment, it significantly decreased to 12.9% (DSS) and 11.1% (DSS + LPL97). The relative abundance of *Mucispirillum* was significantly different between the control and DSS groups (Fig. 13E through I); it increased significantly in the DSS group (1.7%) compared to that in the control group (0.26%), whereas it significantly decreased in the DSS + LPL97 group (0.84%) compared to that in the DSS group (*P* < 0.05). The LPL97 group (0.26%) showed no difference in *Mucispirillum* abundance compared to the control group. *Escherichia-Shigella*, *Staphylococcus*, and *Pseudomonas* also showed the same trend in the control (0.04%, 0.004%, and 0.02%), LPL97 (0.04%, 0.001%, and 0.03%), DSS (0.46%, 0.045%, and 0.07%), and DSS + LPL97 (0.05%, 0.009%, and 0.03%) groups (Fig. 13E through I). The hierarchical clustering heatmap showed the results of distance measurement between clusters at the family and genus levels through Euclidean and Ward parameters (Fig. 14). In the clustering heatmap, the abundance level by taxon indicated the dominant taxon according to the treatment group. The control and the LPL97 groups showed similar taxon clustering and abundance, whereas the DSS and the DSS + LPL97 groups showed unique clustering upon DSS treatment. However, within the unique cluster, the DSS + LPL97 group showed a relatively low abundance compared to the DSS group.

## DISCUSSION

Paraprobiotics are defined as inactivated microbial cells or cell fractions (surface proteins, lipoteichoic acid, peptidoglycans, and exopolysaccharides) that confer health benefits to the host (12), exhibiting immunomodulatory activities and safety advantages *in vitro* (33 – 36). In this study, 11 gut bacteria lysates were screened of 100 lysates based on decreased NO production in RAW 264.7 cells. Oh et al. (37) suggested that inhibition of NO synthesis can ameliorate various inflammatory diseases. We observed that LPL97 treatment effectively reduced (>90%) NO production in RAW 264.7 cells.

*L. petauri* exhibits potential probiotic properties (38). Its genome has functional genes associated with the molecular mechanisms and biochemical processes for these properties (38, 39). We found that the genome of *L. petauri* GB97 carries functional genes for oxygen resistance, arsenic resistance, and intestinal adhesion activities, which may be essential for survival and colonization in the gut habitat (Fig. 2).

*L. petauri* GB97 possesses oxygen resistance-related genes (NADH oxidase, thioredoxin reductase, and SOD) that collectively provide it with temperate oxygen resistance activity, crucial for its survival in the gut environment. These enzymes work together to neutralize H_2_O_2_ and toxic oxygen radicals, converting them into harmless water molecules. Moreover, GB97 has arsenic resistance activity, essential for its survival in the gut habitat, due to the presence of genes encoding arsenate reductase (ArsC), ATP-dependent-arsenite efflux pump (ArsB), and arsenite-stimulated ATPase (ArsA), enabling the conversion and expulsion of arsenic compounds.

Interestingly, GB97 exhibits the potential capacity of sortase-associated modification and activation of cell surface proteins, leading to enhanced bacterial adhesion to the mucus layer and facilitating successful survival and colonization within the gut environment (27). Notably, despite being a gram-positive bacterium, GB97 also harbors *rmlA* and *rmlB* genes, suggesting their participation in the polysaccharide synthesis on the bacterial cell wall (28). In Gram-positive bacteria, the *rmlABCD* gene cluster encodes the four enzymes involved in rhamnose precursor dTDP-L-Rha synthesis (29). According to Sadovskaya et al. (40), some *L. lactis* strains contain rhamnose-rich polysaccharide components underneath the surface-exposed polysaccharide pellicle and trapped inside the peptidoglycan. Conditional mutants producing reduced amounts of rhamnan exhibited strong morphological defects and impaired cell division, indicating that rhamnan is essential for normal growth and division (40). Furthermore, the *rmlABCD* gene cluster has been frequently detected in or around the *epsABCDE* gene cluster of *Lactobacillus* (41). EPS produced by *Lactobacillus* has been associated with immunomodulatory effects (42). This beneficial effect is due to the *rmlABCD* gene cluster producing sugar precursors that are then utilized by the *epsABCDE* gene cluster for EPS biosynthesis (43). Therefore, this specific gene cluster plays a role in EPS biosynthesis and may contribute to further immunomodulatory effects.

Moreover, this genome contains three CpG motifs, probably associated with its immune modulation activity, supporting previously obtained results showing *in vivo* immunomodulation (Fig. S1). CpG motifs can activate host defense mechanisms leading to innate and acquired immune responses (44 – 46). Pretreatment with CpG-oligonucleotides in a DSS-induced colitis mouse model results in increased anti-inflammatory cytokine (IL-10) and decreased pro-inflammatory cytokine (IFN-γ) levels, supporting the immune modulatory effect of CpG motifs (47). Furthermore, the *L. petauri* genome contains the *rmlABCD* gene cluster required to produce sugar precursors for EPS biosynthesis. Therefore, this gene cluster may be associated with the host immunomodulatory effect by EPS.

Herein, LPL97 treatment significantly decreased pro-inflammatory cytokine (TNF-α, IL-1β, and IL-6) levels and increased anti-inflammatory cytokine (IL-10) levels in RAW 264.7 cells. Bacteria lysates exert anti-inflammatory effects by alleviating the inflammation responses in macrophages (40, 48, 49). We observed reduced protein levels of MAPKs (p38, ERK, and JNK), NF-κB (p65 and Iκ-bα), MyD88, and TLR4 upon LPL97 treatment. Surface layer proteins isolated from *Lactobacillus acidophilus* CICC 6074 decreased MAPK and NF-κB levels in RAW 264.7 cells (50). Furthermore, *L. rhamnosus* GG and its components (surface layer protein and exopolysaccharides) can alleviate LPS-induced immune response by suppressing TLR expression and inflammation in porcine intestinal epithelial cells (14). We also observed a reduction in mRNA expression of pro-inflammatory cytokines, iNOS, and COX-2 under LPL97 treatment. These results indicate that LPL97 downregulated inflammation mediators via suppression of the TLR4, MyD88, and NF-κB. and MAPK signaling pathways in LPS-induced RAW 264.7 cells.

We administered 3% DSS with the *L. petauri* GB97 lysate to investigate the probiotic effects of *L. petauri* GB97 in the mouse model. DSS is widely used in the IBD model due to its ability to destroy the epithelial barrier integrity, thus increasing the permeability for DSS or microorganisms (51, 52). The acute phase of DSS-induced colitis involves weight loss, diarrhea, occult blood in stools, and eventually death (51). Body weight is an important indicator of health conditions, and our study showed that DSS treatment leads to weight loss in mice, which was prevented by LPL97 administration. Shortening of the colon is another major symptom of DSS colitis (52), and we observed that LPL97 inhibits reduction in colon length compared with DSS. Histologically, the DSS group showed disruption of epithelial structure and severe mucosal damage, and the LPL97 group showed alleviation of colon tissue lesions. These results are consistent with those of several studies reporting that the gavage of probiotic bacteria lysates ameliorates colitis in mice (18) and rats (53). Chandhni et al. (54) suggested that the surface proteins of probiotic lactobacilli have an ameliorative effect in colitis mouse models. Lipoteichoic acid isolated from *L. acidophilus* regulates colonic inflammation (55). Moreover, probiotics, such as *L. brevis*, *L. plantarum* AR113, and *Bifidobacterium* spp., can alleviate DSS-induced colitis in mice (56 – 58). However, probiotics have safety issues, such as the presence of antibiotic resistance genes (59) and their transfer (60, 61), mucin degradation (62), hemolytic activity (63), and *in vivo* toxicity (64 – 66). Our findings indicated that paraprobiotic LPL97, as an alternative to probiotics, effectively alleviates intestinal inflammation.

The NF-κB signaling pathway plays a key role in the production of inflammation mediators (67). NF-κB activation results in the release of pro-inflammatory cytokines (TNF-α, IL-1β, and IL-6), iNOS, and COX-2 (34, 56). Therefore, inhibition of mRNA and protein expression in the NF-κB signaling pathway is crucial to prevent intestinal epithelial inflammation. In our study, DSS significantly increased the protein expression of NF-κB (p65 and Iκ-bα) compared to the control group, which was significantly reduced by LPL97 and LPL97 + DSS treatments. The mRNA expression of pro-inflammatory cytokines, iNOS, and COX-2, showed consistent protein expression patterns in all groups. However, some differences between mRNA expression and protein levels were observed, particularly for COX-2. These results can be attributed to several factors, including post-transcriptional and post-translational modifications, differences in translation initiation rates, mRNA and protein degradation rates, and other regulatory mechanisms (68 – 70). Nevertheless, we observed that LPL97 modulated the expression of TNF-α, IL-1β, and IL-6, and the secretion of IL-10 in mouse serum, and these results were consistent with our *ex vivo* studies.

The intestinal barrier integrity and mucin layers play an essential role in maintaining intestinal permeability and immune response; they physically protect the host from harmful microorganisms and compounds (71). The maintenance of intestinal barrier function is explained by TJs, which differentiate the apical from the basolateral surface of epithelial cells. The TJ complex consists of transmembrane proteins such as claudin, occludin, junctional adhesion molecules, and intracellular zonulae occludentes (ZOs) (72). The intestine is also covered with a mucin layer, mainly formed of MUC2 released from goblet cells. The mucin layer functions as a physical barrier to protect the intestinal epithelial cells (73). DSS treatment decreased TJ protein (claudin, occludin, and ZO-1) and MUC2 expression in mice (18, 34, 74). Our study also observed downregulated ZO-1, claudin, occludin, and MUC2 mRNA and protein expression levels in the DSS-induced mouse model. These intestinal disorders are promoted by the release of pro-inflammatory cytokines (TNF-α, IL-1β, and IFN- γ) that inhibit the mRNA expression of TJ proteins (75). In this study, LPL97 treatment rescued the DSS-induced decreased mRNA and protein expression levels of TJ proteins, demonstrating that TJ can be restored by suppressing the secretion of pro-inflammatory cytokines through LPL97 treatment.

Intestinal microbial groups play an important role in animal health and growth, including reduced incidence of infection, inflammation, and other immune diseases (76). In this study, microbial changes in the intestines of mice were observed after LPL97 treatment for DSS-induced intestinal inflammation. The most significantly reduced Muribaculaceae in the DSS-treated group was referred to as MIB (mouse intestinal bacteria) as the Bacteroidales order known as S24-7 (77). Known genera belonging to the Muribaculaceae family include *Duncaniella* and *Paramuribaculum*; *Duncaniella* is one of the most dominant and studied S24-7 genera in the mouse gut (78). Although studies on Muribaculaceae that colonize in specific host-derived ecosystems are limited, these genera are major utilizers of monosaccharides, such as O-glycan, in the mouse intestinal mucosa (79) and are greatly affected by DSS, which acts on colonic epithelial cells and impairs mucosal barrier function. *Mucispirillum*, which was significantly increased in the DSS treatment group, is a genus commonly found in mice (80). Although the exact conditions for the proliferation of *Mucispirillum* are still not understood, its abundance indicates enhanced pro-inflammatory responses in the mucosa, accompanied by transiently elevated levels of specific cytokines and amines in plasma (81). Additionally, the pathogenic bacteria, *Escherichia-Shigella*, *Pseudomonas*, and *Staphylococcus*, increased during DSS treatment but tended to decrease in the DSS + LPL97 group. Similar to humans, even in the peritoneal cavity of mice, *Shigella* induces severe diarrhea and acute inflammation in both systemic and mucosal tissues (82). The manifestation of diseases caused by *Shigella* can occur due to reasons such as immunodeficiency or weakened immune response. The invasive pathogen *Shigella flexneri* (*S. flexneri*) is accompanied by the release of inflammatory cytokines IL-1β and IL-18 during the infection stage. The IL-1β signaling pathway induces strong intestinal inflammation, which is a characteristic of *Shigella* infection, and IL-18 is involved in generating an effective antimicrobial response (83). *Staphylococcus* is a well-known bacterium associated with diseases such as sepsis and peritonitis in mice, humans, and livestock (84, 85). *Staphylococcus aureus* (*S. aureus*) can induce intestinal inflammation and diarrhea from overgrowth. *S. aureus* is also associated with IBD due to the ability of gut-derived *S. aureus* antigens to induce inflammatory responses (86, 87). The alpha toxin of *S. aureus* can disrupt barrier function by altering the junctional integrity of intestinal cells *in vitro* (88). *Pseudomonas* causes chronic lung infections, leading to excessive lung tissue remodeling and destruction (89). Gut colonization generally precedes lung infections, and the presence of the same strain in both the gut and lungs suggests a reservoir role of the gut before the transmission of the pathogen to other sites (90 – 92). *Pseudomonas aeruginosa* (*P. aeruginosa*) overgrowth in the gut can lead to bloodstream invasion, posing a serious risk for sepsis development (93). Overall, in our study, DSS-induced acute intestinal inflammation and damage were confirmed in both the DSS and DSS + LPL97 groups, as evidenced by a significant decrease in Muribaculaceae. However, in the presence of immunodeficiency, there was a tendency for decreased abundances of *Mucispirillum*, *Shigella*, *Staphylococcus*, and *Pseudomonas*, which have the potential to induce inflammation in the intestinal mucosa and other tissues, in the DSS + LPL97 group compared to the DSS group. Our results showed that treatment with LPL97 influenced mouse microbial regulation, indicating that it may aid in the prevention and recovery from intestinal inflammation and infection. In addition, oral LPL97 administration did not negatively affect the mouse intestinal microbiota balance.

While our study primarily focused on evaluating the individual effects of LPL97 on inflammation, intestinal barrier function, and gut microbiota composition, it is essential to acknowledge the potential interconnectedness and underlying mechanisms between these factors. A growing body of literature has explored the relationship between lysates from various microbial sources and their impact on these factors. Investigations involving bacterial lysate have demonstrated their immunomodulatory effects in allergic diseases, including atopic dermatitis, allergic rhinitis, and asthma (94). Building upon these studies, our results indicate that LPL97 could influence host health in diverse ways. Although we primarily focused on the effects of LPL97 on inflammation, intestinal barrier function, and gut microbiota composition, *in vivo* and *in vitro* metabolome studies could provide a more profound understanding of the underlying mechanisms of LPL97. The metabolomic alterations due to LPL97 could offer a more comprehensive understanding of its interactions with host systems and their resulting consequences (95, 96). The concept of paraprobiotics, as discussed by Siciliano et al. (97) and Cuevas-González et al. (98), provides valuable insights into the potential therapeutic applications of non-viable microbial cells or their components.

Paraprobiotics have exhibited promising effects in modulating immune responses and maintaining gut homeostasis. Investigating the effects of LPL97 within the context of paraprobiotics would deepen our understanding of its mechanisms of action and its potential as a novel approach for managing inflammation and promoting gut health. Moreover, studies investigating specific cell wall components have highlighted their immunomodulatory effects on immune cells and inflammatory responses. Notably, Kolling et al. (38) explored the immunomodulatory effects of peptidoglycan derivatives from *L. rhamnosus* CRL1505 on respiratory infection in malnourished mice, while Jawhara et al. (99) investigated the anti-inflammatory properties of yeast and cell wall extracts. These studies highlight the potential of cell wall components in modulating immune responses and highlight the importance of understanding the role of LPL97 in this context. Therefore, the effects of LPL97 on inflammation, intestinal barrier function, and gut microbiota composition may be mediated through intricate interactions with the host immune system, epithelial cells, and resident gut microbiota. While our current study does not encompass comprehensive metabolome analysis, its importance is acknowledged. Future studies based on our research should consider metabolomic analyses as part of their experimental design to further elucidate the mechanisms through which LPL97 and other microbial lysates exert their effects. Further investigations into these relationships, including identifying the specific lysate components responsible for its effects, are warranted.

In summary, we isolated an *L. petauri* GB97 cell lysate (LPL97) from healthy porcine feces and evaluated its *in vitro* and *in vivo* anti-inflammatory activities, intestinal barrier function, and gut microbiota changes in LPS-induced murine macrophage and DSS-induced colitis mice. LPL97 regulates immune response by downregulating the TLR4/NF-κB/MAPK signaling pathway *in vitro* and *in vivo*. Furthermore, LPL97 alleviates the disruption of intestinal epithelial integrity and gut microbiota dysbiosis in colitis mice. Therefore, LPL97 has the potential to be developed as an alternative feed additive to antibiotics for the swine industry. However, our study was conducted using mouse models, and further research is necessary to confirm whether the observed protective effects of LPL97 can be replicated in swine studies. Therefore, additional studies focusing on the evaluation of LPL97 in pig models are warranted to assess its potential as an alternative feed additive to antibiotics for the swine industry. Specifically, investigations into the specific components of LPL97 responsible for its anti-inflammatory activity would be valuable.

## MATERIALS AND METHODS

### Isolation and identification of gut bacteria from the porcine stool samples

The stool samples were collected from healthy swine at the National Institute of Animal Science (South Korea). The collected stool samples were suspended in sterilized phosphate-buffered saline (PBS) buffer and serially diluted to 10^−6^. The diluted stool sample was plated on tryptic soy agar containing sterilized 5% sheep blood (TSAB). After incubation in an anaerobic chamber (Coy Laboratory Product, USA) filled with 90% N_2_, 5% CO_2_, and 5% H_2_ at 37°C for 3 days, a single colony was selected and streaked on the fresh TSAB plate. The selected bacterium was identified using 16S rRNA gene sequencing with a universal primer set (27F/1492R). The identified bacterium was stored at −80°C in 10% (wt/vol) sterilized skim milk solution.

### Cell lysate preparation of isolated gut bacteria

Each identified bacterium was inoculated in TSAB at 37°C for 48 h in an anaerobic chamber (Coy Laboratory Product). Cells were washed twp times with sterilized PBS and resuspended with 0.22 mm glass beads. The pellet was lysed using a bead-beater (bioD, South Korea) for 5 min and centrifuged at 12,000 rpm for 15 min at 4°C. The supernatant was collected and filter-sterilized using a 0.2-µm syringe filter (Millipore, USA). Protein concentrations were determined using a Pierce BCA protein assay kit (Thermo Scientific, USA). Gut bacteria lysates were diluted with 20 µg/mL of total protein in PBS and stored at −80°C until use.

### NO production determination of selected gut bacteria

#### Treatment of RAW 264.7 cell line with bacterial cell lysate and LPS

RAW 264.7 cells (Korean Cell Line Bank, South Korea) were seeded into 24-well culture plates (Corning, USA) at 2.2 × 10^5^ cells/well and incubated in Dulbecco’s modified Eagle’s medium (DMEM; Gibco, USA) supplemented with 10% heat-inactivated fetal bovine serum (FBS; Gibco) and 1% (vol/vol) antibiotics (100 U/mL penicillin and 100 µg/mL streptomycin) at 37°C in 5% CO_2_ incubator for 24 h. Confluent macrophage cells were treated with the cell lysate of 100 isolated gut bacteria for 4 h, and then 100 ng/mL LPS-derived from *E. coli* O55:B5 (Sigma-Aldrich, USA) was added to each well, and the culture plates were incubated at 37°C for 20 h. All supernatant samples were collected (2,000 rpm, 5 min, 4°C).

#### NO assay

To determine the NO concentration, a 100 µL of Griess reagent [2% sulfanilamide (wt/vol) in 5% phosphoric acid, 0.2% *N*-(1-Naphthyl) ethylenediamine dihydrochloride in H_2_O (wt/vol) (1:1)] (100) was added to 100 µL of a collected supernatant sample. After 15 min reaction at room temperature, the optical density of the solution at a wavelength of 540 nm was determined using a microplate reader (Varioscan Flash; Thermo Fisher Scientific). The concentration of NO was determined by converting the level of OD_540_ to the concentration using a standard curve of NaNO_2_.

### Whole-genome sequencing and bioinformatic analysis

#### Genomic DNA extraction

The genomic DNA of *L. petauri* GB97 was extracted using the G-spin Genomic DNA Extraction Kit, according to the manufacturer’s instructions (Intron Biotechnology, South Korea). After extraction, the purity and quantity of genomic DNA were determined using a NanoDrop 2000 Spectrophotometer (Thermo Fisher Scientific).

#### Genome sequencing and bioinformatics

The extracted genomic DNA was sequenced using the Illumina MiSeq system (Sanigen, South Korea). After genome sequencing, the qualified raw sequence reads were used for genome assembly using Unicycler v0.4.8 (101). ORF prediction and gene annotation were performed using Prokka v1.12 (102). Functional analysis of predicted ORFs was performed using the InterProScan program (103). COG functional categorization and metabolic pathway analysis were conducted with COGNIZER and KASS, respectively (104, 105). A circular genome map was generated using GenVision (DNASTAR, USA), and CpG motifs were predicted using CpGFinder with default parameters (Softberry, USA).

#### Phylogenetic analysis

ANI values were calculated using JSpecies v1.2.1 based on the BLAST algorithm (NCBI, USA). The ANI-based phylogenetic tree was constructed with the calculated ANI values and visualized using the RStudio software of the R package (106).

### Safety assessment of *L. petauri* GB97

#### Prediction of virulence factors and antibiotic resistance genes

Prediction and analysis of virulence and pathogenicity factors were performed with VFDB (107) and PathogenFinder 1.1 (108). In addition, CARD (109) and ResFinder 3.0 (110) were used for identifying the antibiotic resistance genes and their transfer.

#### Hemolytic activity

Hemolytic activity test was performed according to the blood agar plates and hemolysis protocols of the American Society for Microbiology (111).

#### Lactate dehydrogenase assay

The human colorectal cancer cell line, Caco-2 (Korean Cell Line Bank), was cultured in DMEM (Gibco) supplemented with 10% heat-inactivated FBS (Gibco) and 1% (vol/vol) antibiotics (100 U/mL penicillin and 100 µg/mL streptomycin) at 37°C in 5% CO_2_ incubator. Cells (1 × 10^4^ cells/well) were seeded in 96-well culture plates (SPL, South Korea) in a growth medium. After 24 h of seeding, *L. petauri* GB97 (10^7^, 10^8^ CFU/well) were added and incubated at 37°C in 5% CO_2_ for 24 h. Lactate dehydrogenase (LDH) released by the damaged cells was measured using an LDH assay kit (DoGenBio, South Korea). PBS was used as a negative control and *E. coli* O157:H7 ATCC 43895 was used as a positive control. The results were expressed as a percentage of cytotoxicity (%) (112).

### Anti-inflammation test

#### RNA extraction and real-time qPCR

Total RNA was isolated from RAW 264.7 cells using the RNeasy Mini kit (Qiagen, Germany) using the manufacturer’s standard protocol. RNA quality and quantity were determined using a NanoPhotometer (Implen, Germany). Extracted RNA was reverse-transcribed to generate complementary DNA (cDNA) using the AccuPower CycleScript RT premix (Bioneer, South Korea) according to the manufacturer’s instructions. RT-qPCR was conducted using a StepOnePlus Real-Time PCR System (Thermo Scientific) with SYBR Green PCR Master Mix (Thermo Scientific). The RT-qPCR reaction was performed in a 96-well plate containing 10 mL of SYBR green PCR master mix, 2 µL of primers (5 pmol), 2 µL of cDNA template, and 6 µL of nuclease-free water. The RT-qPCR conditions were as follows: 1 cycle of 95°C for 10 min; 40 cycles of 95°C for 15 s, 60°C for 1 min, and 72°C for 40 s. The relative gene expression was determined by using the 2^–ΔΔCT^ comparative method (113). All data were normalized to the expression level of the β-actin housekeeping gene.

#### Cytokine assay

To determine mRNA expression and protein levels of pro-inflammatory and anti-inflammatory cytokines, four groups (control, LPS, LPL97, and LPL97 + LPS) were prepared. The control group is RAW 264.7 cells treated with PBS buffer; the LPS group is the cells treated with 100 ng/mL LPS per well; the LPL97 group is the cells treated with 20 µg/mL of *L. petauri* GB97 lysate (LPL97) per well; and the LPS + LPL group is the cells treated with 100 ng/mL LPS and 20 µg/mL of *L. petauri* GB97 lysate per well. The mRNA expression levels of pro-inflammatory cytokines (TNF-α, IL-6, and IL-1β) and anti-inflammatory cytokine (IL-10) from RAW 264.7 cells in those four groups were quantified using RT-qPCR as described above. Cytokine-specific primer sets are listed in Table 2. Inflammatory cytokines from RAW 264.7 cells in those four groups were quantified using Cytokine ELISA kits (Invitrogen, USA) according to the manufacturer’s instructions.

#### Western blot analysis

RAW 264.7 cells were rinsed twice with ice-cold PBS and lysed in RIPA buffer (Thermo Fisher Scientific) with phosphatase and protease inhibitor cocktail (Sigma-Aldrich). It was stored on ice for 5 min and centrifuged at 12,000 rpm at 4℃ for 15 min. After centrifugation, the supernatant was collected, and the protein concentration was measured by a BCA assay kit (Thermo Fisher Scientific). About 20 µg of protein samples was separated using SDS-PAGE analysis with 4–12% Bolt Bis-Tris gel (Thermo Fisher Scientific). The separated protein bands were then transferred onto polyvinylidene difluoride (PVDF) membranes (Bio-Rad Laboratories, USA) at room temperature for 1 h. The membranes were incubated with rabbit primary antibodies (Table S1) overnight at 4℃. After antibody hybridization, the membranes were washed four times with TBST (25 mM Tris HCl, 0.15 M NaCl, and 0.05% Tween 20) to remove all excess primary antibodies and incubated again with anti-rabbit horseradish peroxide conjugated secondary antibodies (1:5,000 dilution, Cell Signaling, USA) at room temperature for 1 h. The target protein was visualized and detected with enhanced chemiluminescence (ECL) reagent (Enzynomics, South Korea) using the Davinci-Chemi Imaging System (Davinci-K, South Korea).

### *In vivo* anti-inflammation test using a colitis mouse model

All animal experiments were approved by the Institutional Animal Care & Use Committee (Dankook IACUC, Cheonan, South Korea; approval no. DKU-21-039). Seven-week-old male C57BL/6 mice were purchased from Raon Bio Inc. (South Korea). The environment was maintained at 22 ± 2°C and 24 ± 2% relative humidity under 12 h alternating lighting cycles supplied with sufficient water and basal diet. The C57BL/6 mice had a week-long adaption period under the experimental conditions and then were randomly divided into four groups (*n* = 3): control (1× PBS, per oral), DSS (3% DSS in the water supply), LPL97 (*L. petauri* GB97 lysate; 10^9^ CFU/mL in 1× PBS, per oral), and LPL97 + DSS (*L. petauri* GB97 lysate; 10^9^ CFU/mL in 1× PBS per oral and 3% DSS in the water supply; Fig. 6A).

The total feeding study was performed for a total of 2 weeks. The control group was treated once a day with 100 µL of 1× PBS (weeks 0–2); The DSS group received 1× PBS for the first week, but in the second week, they were given 3% DSS (molecular mass 36–50 kDa, MP Biomedicals, USA) dissolved completely in the drinking water; the LPL97 group was fed once a day with 100 µL of LPL97 (10^9^ CFU/mL in 1× PBS) for 2 weeks orally; the LPL97 + DSS group was administered with 100 µL of LPL97 once a day for the first week, but in the second week, they were given LPL97 orally and 3% DSS solution (Fig. 6A).

During this study, the body weight of the mice was measured every day, along with the body weight change (%). After the feeding study, the blood samples were collected from the orbital blood vessels and centrifuged at 4,000 rpm at 4°C for 20 min for serum collection. The collected serum samples were stored at −80°C until further analysis. The mice were euthanized by cervical dislocation and then dissected. The colon lengths were measured, and the middle part of the colon was fixed with 10% (vol/vol) of neutral buffered formalin (Sigma-Aldrich) for hematoxylin and eosin staining. The rest of the colon tissues were washed with cold PBS and stored at −80°C for RT-PCR and western blot analysis. Fecal samples of all mice were collected into sterilized Eppendorf tubes and stored at −80°C for gut microbiota analysis.

The colon tissues fixed with a 10% formalin solution were embedded in paraffin and sectioned using standard protocols (112). Tissue sections were mounted on glass slides, deparaffinized, and stained with hematoxylin and eosin for histological analysis. Images were obtained from a light microscope (Olympus BX41; Olympus Optical, Japan).

To analyze the inflammatory cytokines in the four groups (control, DSS, LPL97, and LPL97 + DSS), their mRNA expression levels in the colon tissues were measured using RT-qPCR following the protocol described in the “RNA extraction and real-time qPCR” section, above. Western blot analysis was performed using tissue lysates, and each condition was run on the same gel. The actin loading control was employed across all conditions to ensure consistency in the experiments. Additionally, the levels of cytokines in the serum were measured using the ELISA method following the protocol in the “Cytokine assay” section, above.

### Gut microbiota analysis

Fecal samples were collected at week 0 (before feeding started) and week 2 (after feeding stopped) from four groups (3 mice per group; control, DSS, LPL97, and LPL97 + DSS). Total DNA was extracted from 200 mg of collected feces per sample using the QIAamp Fast DNA Stool Mini Kit (Qiagen, Germany) based on the manufacturer’s manual. DNA concentrations were measured using a Colibri Microvolume Spectrometer (Titertek-Berthold, Germany), and only qualified DNA samples with an OD_260/280_ ratio between 1.85 and 2.15 were used for further analysis. 16S rRNA amplicons were prepared with a V5–V6 targeting universal primer set (799F-mod6/1114R) under the following PCR condition: one cycle of 98℃ for 3 min, 30 cycles of 98℃ for 10 s, 57℃ for 5 s and 68℃ for 1 s, and one cycle of 72℃ for 5 min. After 16S rRNA PCR, the amplicons were purified using Wizard SV Gel and PCR Clean-Up System (Promega, USA). The NGS sequencing was conducted with Illumina MiSeq System at Cancerrop (South Korea). MicrobiomeHelper software package was used to identify gut bacteria and Quantitative Insights into Microbial Ecology 2 (QIIME2) was used to analyze microbial diversity from the 16S rRNA amplicon sequences. Beta diversity was measured using the weighted (quantitative) and unweighted (qualitative) UniFrac distance metrics. The naïve Bayesian classifier-trained RDP reference database was used for the taxonomic assignment. Using the Euclidean and Ward parameters, clusters were designated by measuring the distance between data points and the distance between clusters. The 16S rRNA gene raw data have been uploaded to the Sequence Read Archive public repository of the National Center for Biotechnology Information (accession number: PRJNA982390).

### Statistical analysis

Statistical significance was calculated using the Student’s two-tailed *t* test within the Statistical Package for Social Sciences (SPSS 22.0; IBM, USA). Results were expressed as the mean ± SD, and differences between means were considered significant at *P* < 0.05. The gut microbiome parameters were calculated using GraphPad Prism version 7.00 for Windows (GraphPad Software, USA). A Kruskal-Wallis *H*-test was used to confirm the data from alpha diversity analysis, including observed features, Shannon, Chao1, and Simpson indices. ANOSIM was used to determine whether the microbial compositions among the four groups were significantly different using QIIME2. To identify significant differences in the relative abundance of microbial taxa among the four groups, the Tukey-Krammer multiple comparison test (two-way analysis of variance) was used in the Statistical Analysis of Metagenomic Profiles (STAMP) software v2.1.3. Statistical significance was set as *P* < 0.05.

## Data Availability

The high-throughput sequencing data generated in this study have been deposited in the NCBI SRA database under the accession number PRJNA982390. The GenBank accession number for the nucleotide sequence of *L. petauri* GB97 is CP127854.

## SUPPLEMENTAL MATERIAL

Supplemental figure captions spectrum.01334-23-s0001.docx Captions of Fig. S1 and S2.

Figure S1 spectrum.01334-23-s0002.tif CpG motifs in the *L. petauri* GB97 genome sequences. The vertical axis refers to the GC percentage (black, left) and observed/expected ratio (blue, right). The purple areas represent CpG motifs I, II, and III. The red vertical lines indicate CpG dinucleotides.

Figure S2 spectrum.01334-23-s0003.tif Assessment of LPL97 cytotoxicity in RAW 264.7 cells using CCK-8 (A) and Trypan Blue (B) assays.

Table S1 spectrum.01334-23-s0004.docx List of antibodies used in this study.

ASM does not own the copyrights to Supplemental Material that may be linked to, or accessed through, an article. The authors have granted ASM a non-exclusive, world-wide license to publish the Supplemental Material files. Please contact the corresponding author directly for reuse.
