# Supplementary material for: Characteristics of Lactococcus petauri GB97 lysate isolated from porcine feces and its in vitro and in vivo effects on inflammation, intestinal barrier function, and gut microbiota composition in mice
Source: Microbiol Spectr. 2023 Nov 29;12(1):e01334-23. doi: 10.1128/spectrum.01334-23 (PMC10782967; doi:10.1128/spectrum.01334-23)
Supplement: Supplemental figure captions — Captions of Fig. S1 and S2. [file spectrum.01334-23-s0001.docx]

**Figure S1**. CpG motifs in the *L. petauri* GB97 genome sequences. The vertical axis refers to the GC percentage (black, left) and observed/expected ratio (blue, right). The purple areas represent CpG motifs I, II, and III. The red vertical lines indicate CpG dinucleotides.

**Figure S2**. Assessment of LPL97 cytotoxicity in Raw 264.7 Cells Using CCK-8 (A) and Trypan Blue (B) Assays.
