# Supplementary figures and images for: Characteristics of Lactococcus petauri GB97 lysate isolated from porcine feces and its in vitro and in vivo effects on inflammation, intestinal barrier function, and gut microbiota composition in mice
Source: Microbiol Spectr. 2023 Nov 29;12(1):e01334-23. doi: 10.1128/spectrum.01334-23 (PMC10782967; doi:10.1128/spectrum.01334-23)

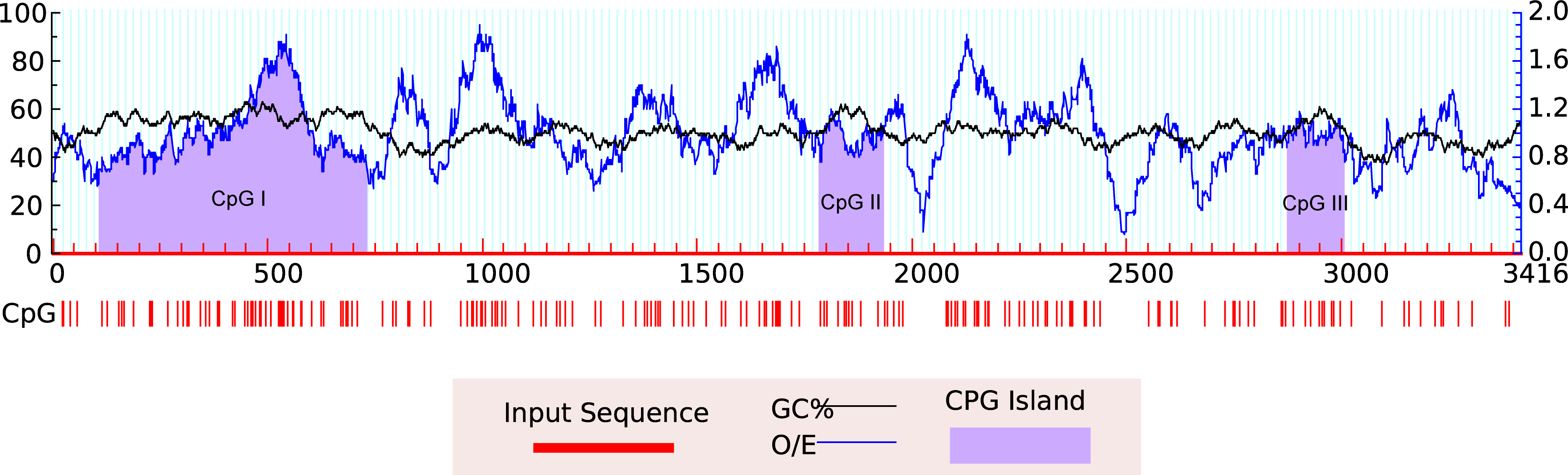

Supplement: Figure S1 — CpG motifs in the L. petauri GB97 genome sequences. The vertical axis refers to the GC percentage (black, left) and observed/expected ratio (blue, right). The purple areas represent CpG motifs I, II, and III. The red vertical lines indicate CpG dinucleotides. [file spectrum.01334-23-s0002.tif]

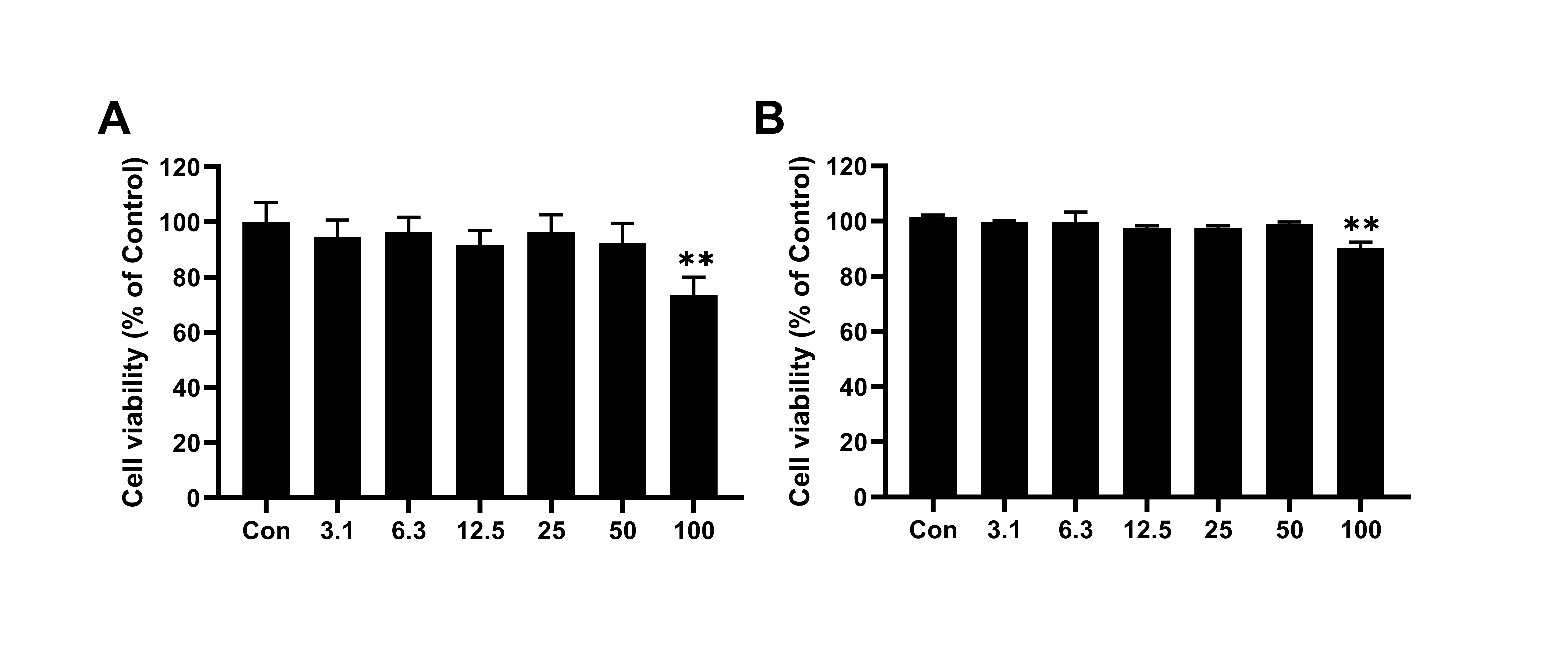

Supplement: Figure S2 — Assessment of LPL97 cytotoxicity in RAW 264.7 cells using CCK-8 (A) and Trypan Blue (B) assays. [file spectrum.01334-23-s0003.tif]
