# Supplementary material for: Characteristics of Lactococcus petauri GB97 lysate isolated from porcine feces and its in vitro and in vivo effects on inflammation, intestinal barrier function, and gut microbiota composition in mice
Source: Microbiol Spectr. 2023 Nov 29;12(1):e01334-23. doi: 10.1128/spectrum.01334-23 (PMC10782967; doi:10.1128/spectrum.01334-23)
Supplement: Table S1 — List of antibodies used in this study. [file spectrum.01334-23-s0004.docx]

**Table S1**. List of antibodies used in this study.

| Antibody | Company | Dilution |
| --- | --- | --- |
| Toll-like Receptor 4 Rabbit mAb  (Cat number: #14358) | Cell Signaling Technology, Danvers, MA, USA | 1:1,000 |
| iNOS Rabbit mAb (Cat number: #13120) | Cell Signaling Technology | 1:1,000 |
| Cox2 Rabbit mAb (Cat number: #12282) | Cell Signaling Technology | 1:1,000 |
| NF-κB p65 Rabbit mAb (Cat number: #8242) | Cell Signaling Technology | 1:1,000 |
| Phospho NF-κB p65 Rabbit mAb (Cat number: #3033) | Cell Signaling Technology | 1:1,000 |
| Iκ-bα Rabbit mAb (Cat number: #2859) | Cell Signaling Technology | 1:1,000 |
| Phospho Iκ-bα Rabbit mAb (Cat number: #9165) | Cell Signaling Technology | 1:1,000 |
| MyD88 Rabbit mAb (Cat number: #4283) | Cell Signaling Technology | 1:1000 |
| p38 MAPK Rabbit mAb (Cat number: #8690) | Cell Signaling Technology | 1:1,000 |
| Phospho p38 MAPK Rabbit mAb (Cat number: #4511) | Cell Signaling Technology | 1:1,000 |
| SAPK/JNK MAPK Rabbit mAb (Cat number: #9252) | Cell Signaling Technology | 1:1,000 |
| Phospho SAPK/JNK MAPK Rabbit mAb (Cat number: #9251) | Cell Signaling Technology | 1:1,000 |
| p44/42 MAPK Rabbit mAb (Cat number: #4695) | Cell Signaling Technology | 1:1,000 |
| Phospho p44/42 MAPK Rabbit mAb (Cat number: #9101) | Cell Signaling Technology | 1:1,000 |
| β-actin Rabbit mAb (Cat number: #8457) | Cell Signaling Technology | 1:1,000 |
| ZO-1 Polyclonal Antibody (Cat number: #40-2200) | Invitrogen, Carlsbad, CSA, USA | 1:1,000 |
| Occludin Polyclonal Antibody (Cat number: #34-1700) | Invitrogen | 1:1,000 |
| Claudin-3 Polyclonal Antibody (Cat number: #40-4700) | Invitrogen | 1:1,000 |
| Anti-rabbit IgG, HRP-linked Antibody (Cat number: #7074) | Cell Signaling Technology | 1:5,000 |
